# Spatial distribution and pollution evaluation in dry riverbeds affected by mine tailings

**DOI:** 10.1007/s10653-022-01469-5

**Published:** 2023-01-16

**Authors:** J. G. Cuevas, A. Faz, S. Martínez-Martínez, M. Gabarrón, J. C. Beltrá, J. Martínez, J. A. Acosta

**Affiliations:** Sustainable Use, Management and Reclamation of Soil and Water Research Group, Universidad Politécnica de Cartagena, Paseo Alfonso XIII 48, 30203 Cartagena, Spain

**Keywords:** Mar Menor lagoon, Sediments, Metals, Pollution index, Multivariate statistical analysis

## Abstract

The objective of this study was to evaluate the level of pollution, sources and potential risk of heavy metals (Zn, Cu, Mn, Cd, Cr, Ni, Fe and Pb) and arsenic (As) in four dry riverbeds affected by mine tailing, which drain into one of the biggest coastal lagoon of Europe (Mar Menor). El Beal, La Carrasquilla, Las Matildes and Ponce dry riverbeds sediments were sampled along its course (20, 18, 13, 19 samples were collected, respectively), and total/soluble metal(loid)s, water soluble ions, nitrogen, and organic/inorganic carbon contents were analyzed. Spatial distribution, principal component analysis (PCA), hierarchical cluster analysis (HCA), contamination factor (Cf), pollution load index (PLI) and potential ecological risk index (RI) were used to identify the possible sources of metal(loid)s and to assess the sediment pollution status. The results showed that the mean total concentrations of As, Cu, Cd, Mn, Zn and Pb exceeded the natural background levels of the study area, with the highest values located close to the mining areas. Correlation and cluster analysis identified that Cd and Zn were associated mainly with anthropogenic activities for all riverbeds, while Cr and Ni come from parent. PLI graded the four riverbeds as contaminated by heavy metals, while RI manifested that 100% of samples located in El Beal, La Carrasquilla and Las Matildes had a significantly high ecological risk. Therefore, this study suggests that mine wastes are the main source of metal(loids) contamination in the dry riverbeds, which results can be used to design actions and measures to reduce the environmental impact of metal(loid)s in the Mar Menor coastal lagoon.

**Supplementary Information:**

The online version contains supplementary material available at 10.1007/s10653-022-01469-5.

## Introduction

Metal mining produces a large amount of waste with a high concentration of metals and arsenic, that can be mobilized into the environment during the exploitation period and after ceasing mining activities (Cappuyns et al., 2006). These potential toxic elements could be released into the environment through weathering and leaching processes, becoming an important source of environmental pollution (Gonzalez-Fernandez et al., 2011; Montofré et al., 2021). The mobility of metal(loid)s depends on several factors such as pH, redox potential, amount of organic matter and ion exchange processes (Filgueiras et al., 2004), which can occur on the surface of waste dumps, ponds, and slag heaps, representing the most persistent environmental impact from the mining industry (Gundersen et al., 2001).

Rivers flowing from the mining areas to the sea can transport mine waste enriched on metal(loid)s, become a pollution source, where metal(loid)s constitute one of the most dangerous transported elements due to their toxicity, tendency to accumulate in organisms and nondegradable chemical properties (Fufeyin & Egborge, 1998). As metal(loid)s cannot be degraded, they are deposited, assimilated, or incorporated in water, sediment and aquatic animals (Linnik & Zubenko, 2000). Sediments, which are an important part of these ecosystems, act as storage for metal(loid)s, where large concentrations of these elements are ultimately incorporated into their composition (Rolfe and Edgiston, 1973). In addition, soils of the riverbanks are also impacted by wastes washed downstream resulting in low pH, low organic matter and scarce or null vegetation (Conesa et al., 2006). Metal(loid)s pose a serious risk to human health for being nondegradable and can be bioaccumulated by plants that can translocate them to its edible organs (Wong, 2003). In addition, in the human metabolism metal(loid)s can be biomagnified via the food chain and finally assimilated resulting in health risks (Agah et al., 2009).

Climatic effects like wind storms and heavy rainfalls have a greater impact on the dispersion of metals in Mediterranean areas since the soils are typically sparely vegetated (Kempton & Atkins, 2000). Wind erosion can be a major cause of the loss and dispersion of waste material from mine areas, inducing fine particle dispersion (Blight, 2008). In addition, heavy rainfalls remove and transport particles from tailings to the dry riverbeds and promote the formation of acid mine drainage (AMD) (Caraballo et al., 2016).

To assess the risk of metals in soil, a variety of methods have been used, including methods to interpret the degree of contamination in soils based on background levels, being the most frequently used indices the contamination factor (Cf), pollution load index (PLI), and potential ecological risk index (RI) (Enuneku et al., 2017; Lam et al., 2020, 2022). The limitations of these geochemical approaches are often satisfied with the use of multivariate chemometric techniques that include principal component analysis (PCA) and cluster analysis (CA) (Kowalska et al., 2018). Furthermore, understanding the spatial distribution of metal(loid)s concentration along the dry riverbeds is an important task for researchers and policy makers to implement regulatory actions to reduce potential risks and prioritize intervention areas.

Historically Cartagena-La Union mining district contained important lead (Pb)–zinc (Zn) massive ore deposits; however, mining activities ceased in 1991. Exploited ores mainly consisted of the exploitation of galena (PbS), sphalerite (ZnS), and pyrite (FeS_2_) (Alcolea-Rubio, 2015). The implementation of flotation techniques in the 50´s promoted a huge increase in landfill disposal sites along the area (Martinez, 2005). Mining wastes were deposited in different ways along the years, initially, they were directly thrown into dry riverbeds or conducted by pipes into the sea causing a great environmental impact, to remediate this situation, the Government of Spain in 1955 prohibited these practices, forcing companies to deposit the mine wastes in mining dams forming mine ponds (Martínez-Sánchez et al., 2008). However, the coastal area is been currently affected by direct or indirect discharges (Khademi et al., 2018).

Mar Menor coastal lagoon is one of the largest coastal lagoons in the Mediterranean region, where four dry riverbeds pour their waters into the lagoon. Currently, those riverbeds remain dry for long periods, where sediments and water do not reach the lagoon unless sporadic and torrential rainfall occurs. However, in a semi-arid Mediterranean context, violent climatic events, such as high-speed winds and heavy rainfall can occur in a very short time frame (Boussen et al., 2013). The hydrological regime makes these four riverbeds very important in the mobilization and transport of contaminants, collecting the surface runoff to the coastal.

Therefore, the objectives of this study were: (1) to determine the concentrations and spatial distribution of cadmium, chromium, copper, iron, manganese, nickel, lead, zinc and arsenic in the sediments from the four dry riverbeds (2) to assess sediments pollution status and their potential hazards to the environment using different pollution indices, and (3) identify possible sources of the metal(loid)s in the sediments by using multivariate statistical analysis.

## Materials and methods

### Study area

Sierra Minera Cartagena-La Unión is one of the oldest mining districts in Europe, located in the inner zone of the Betic mountain range (SE, Spain), where metal mining was developed since Phoenician and Carthaginian times until its closure in 1991 (González-Fernández et al., 2008). Iron is present as oxides, hydroxides, sulfides, sulfates, carbonates and silicates; Pb and Zn occur as galena and sphalerite, respectively, and in form of sulfate, carbonates and oxides (Oen et al., 1975). As a consequence of this activity, in this region, there are more than 85 mining ponds of waste from Pb and Zn sulfides, and therefore, richness in toxic elements such as As, Pb, and Cd. The main metallics composition of the ponds are Fe, Pb and Zn, with lower concentrations of As and Cd (Alcolea, 2015). The semi-arid climate of the area has an annual rainfall of about 250–300 mm, concentrated in spring and autumn, where torrential rains usually happened. Highlight that in September 2009, the largest amount of accumulated precipitation in a single downpour was recorded (335.2 mm) (Betancourt-Suárez et al., 2021). In addition, the maximum daily rainfall in 2019 was 217.8 mm from September 12 (12:00 h) to September 13 (12:00 h) corresponded to a 500-year return period (Erena et al., 2020). The average annual temperature is 18 °C (Conesa et al., 2008).

**Fig. 1:**
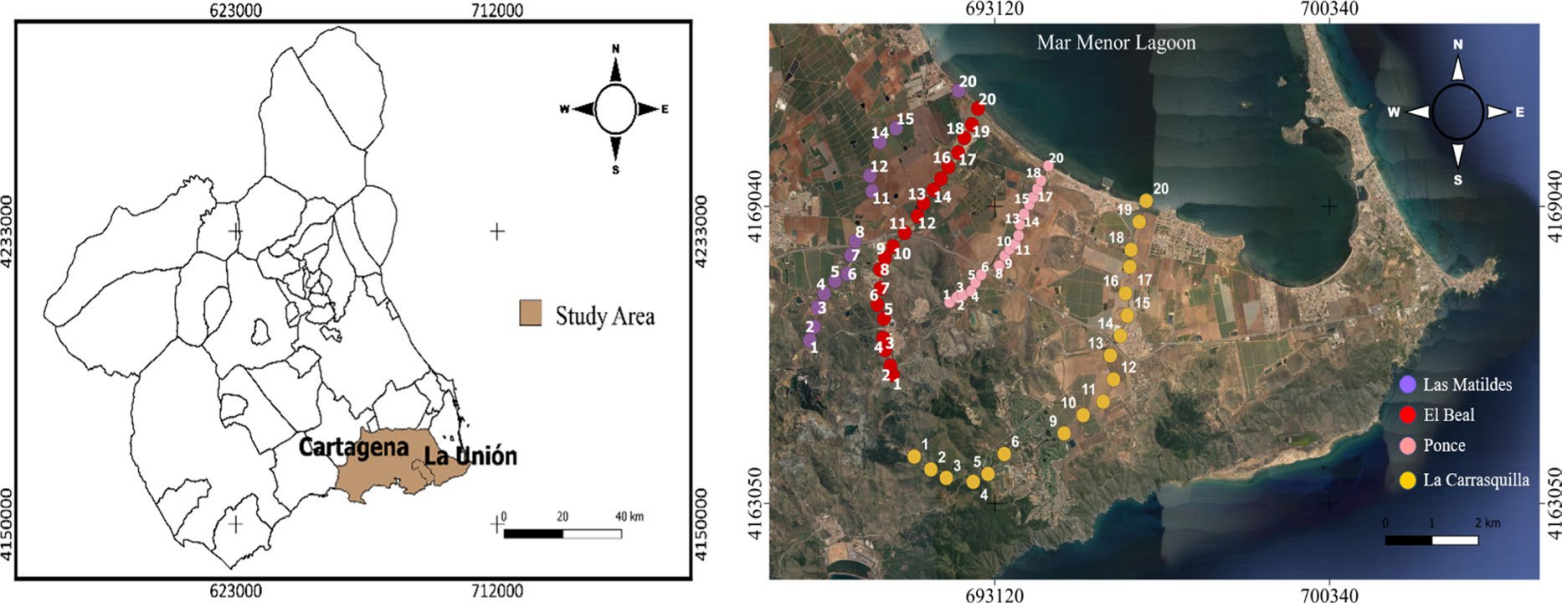
Sampling points in dry riverbeds located in the Cartagena-La Unión mining district

In this area, there are unconnected dry riverbeds, a system that functions separately with its drainage points, which are responsible of Mar Menor coastal lagoon contamination from the mining area (Fig. 1). The distribution of mining-metallurgical wastes is 60% of the total volume located in the basins of dry riverbeds dumped into Mar Menor lagoon, and the remaining 40% is located in the basins dumped into the Mediterranean Sea (García, 2004). This coastal area concentrates metal(loid)s in the sediments, a phenomenon that extends to its shores and associated wetlands, considered with a great ecological value (Baraza, 2003). The deposition of metals in the riverbeds has caused the appearance of different sedimentary levels with a great variety in terms of chemical properties, where variability depends on the origin of these sediments and the composition of the wastes that were released (González-Fernández et al., 2008). Therefore, the geochemical characterization of the riverbeds sediments is an essential tool that will allow to determine the distribution of heavy metals along the riverbeds and to identify their sources, also it can be used to evaluate the environmental risk as for people as for the ecosystems in contact with these dry riverbeds.

El Beal (EB) riverbed has a surface area of 7.60 km^2^, a perimeter of 17.3 km, and a bed length of 7.20 km, the height difference is 242 m with an average slope of 1.90%. La Carrasquilla (LC) has a surface area of 29.0 km^2^, a perimeter of 25.8 km, and a bed length of 10.6 km, the height difference is 232 m with an average slope of 1.30%. Las Matildes (LM) has a surface area of 17.4 km^2^, a perimeter of 26.2 km, and a bed length of 6.73 km, the height difference is 379 m with an average slope of 3.20%. Lastly, Ponce (PN) riverbed has a surface area of 11.9 km^2^, a perimeter of 16.6 km, and a bed length of 6.40 km, the height difference is 384 m with an average slope of 3.10% (Garcia, 2004).

### Sampling and analytical methods

For the four dry riverbeds studied, Beal (EB), Carrasquilla (LC), Matildes (LM) and Ponce (PN), 20, 18, 13 and 19 sediment samples were collected, respectively. The sampling points were distributed covering the entire surface of the riverbeds from the mine area to the river mouth. Three soil samples (0–30 cm depth) were collected with a soil spade on each point and then mixed to get a composite sample. Then, soil samples were packed and sealed in polyethylene bags and transported to the laboratory for analysis. The samples were dried in an oven at 40ºC for 72 h and passed through a 2 mm sieve. The pH was measured for a soil:water ratio of 1:2.5 while the electrical conductivity (EC) was measured in aqueous extract 1:5 (weight:volume) (Soil Survey Division Staff, 1993).

Water soluble calcium (Ca^2+^), magnesium (Mg^2+^), sodium (Na^+^), chloride (Cl^−^), nitrate (NO_3_^−^), and sulfate (SO_4_^2−^) were measured by 861 METROHM ion chromatography system, using deionized water in a 1:5 soil:water ratio. Total carbon and nitrogen were determined using CHN 628 DE LECO, soil inorganic carbon was measured by the modified Pressure Calcimeter Method with a Bernad Calcimeter (Porta et al., 1986). Soil organic carbon was calculated from the difference between total and inorganic carbon.

The concentrations of arsenic (As), cadmium (Cd), chromium (Cr), copper (Cu), iron (Fe), manganese (Mn), nickel (Ni), lead (Pb), and zinc (Zn) were determined by acid digestion, 0.5 g of ground soil was weighed, then digested in teflon vessels with nitric acid (HNO_3_) and hydrochloric acid (HCl) (US-EPA 3051) in a microwave (Acosta et al., 2018). Subsequently, the samples were filtered and the concentration of metals and arsenic were determined by inductively coupled plasma spectrometer (ICP-MS PerkinElmer optima 8300-DV) (EPA, 1996). Certified reference material (BAM-U110) from the Federal Institute for Materials Research and Testing (F.I.M.R.T., 2010) and reagent blanks were used as the quality control samples during the analysis. The recovery of metals in the analysis was within < 5.0% compared to this reference sample.

### Quality indices for pollution evaluation

Quality indices indicate the level of a parameter compared to a respective classification indicating a low, moderate, or high degree of contamination (Rees et al., 2008).

#### Contamination factor

Contamination factor (Cf) (Eq. 1) establishes the degree of contamination of an individualized element, using background concentration as a starting point (Hakanson, 1980).

1 $${\text{Cf}} = \frac{{C_{{\text{Sample }}} }}{{C_{{\text{Background }}} }}$$

where *C*_sample_ is the mean metal(loid) concentration in the sample, and *C*_background_ is the background concentration in the area (Table 1).

**Table 1:** Local geochemical background (mg kg^−1^) metal(loid)s concentration (Martínez Sánchez et al., 2007)

As	Cd	Cr	Cu	Ni	Mn	Pb	Zn
7	0.32	40.4	12.6	21.7	770^*^	9.30	41.4

^*^Mn background established by Ministére de l´Environmement du Québec (2001)

#### Pollution load index

Pollution load index (PLI) was proposed by Tomlinson et al. (1980), it is used to determine the integrated degree of contamination of all the metals analyzed (n). PLI (Eq. 2) is an empirical index that provides a simple and comparative means to evaluate the level of metal contamination (Mohammad et al., 2010).

2 $${\text{PLI}} = n\sqrt {{\text{Cf}}_{{{\text{i}}1}} {\text{ xCf}}_{{{\text{i}}2}} {\text{x Cf}}_{{{\text{i}}3}} {\text{ x}} \ldots {\text{Cf}}_{{{\text{in}}}} }$$

where Ci_f1_ is the contamination factor of each metal(loid) (Eq. 1). If the PLI shows a result higher than 1, the sample is contaminated (Varol, 2011).

#### Potential ecological risk index

Potential ecological risk (RI) was developed by Hakanson (1980). RI (Eq. 3) was introduced to assess the degree of trace metal contamination based on the toxicity of each metal. The index does not assess the magnitude of anthropogenic change, but provides the potential for biological uptake that may affect organisms in the ecosystem (Birch, 2016).

3 $$RI = \mathop \sum \nolimits_{i = 1}^{n} {\text{E}}_{{{\text{ri}}}}$$

4 $${\text{E}}_{{{\text{ri}}}} = {\text{T}}_{{{\text{ri}}}} {\text{ X Cf}}_{{\text{i}}}$$

where ${\mathrm{Cf}}_{\mathrm{i}}=\frac{{\mathrm{C}}_{\mathrm{Si }}}{{\mathrm{C}}_{\mathrm{ni}}}$, C_si_ is the concentration of the element in question present in the sample and C_ni_ is the background value of the element in each sample. E_ri_ (Eq. 4) corresponds to the RI of an individual element, hence RI is the sum of E_ri._ and T_ri_ is the biological toxicity factor of an individual element, which has set for Cd, Cr, Cu, Mn, Ni, Pb, Zn and As these values: 30, 2, 5, 1, 5, 5, 1, and 10, respectively (Yi et al., 2011).

The classification of the degree of contamination according to the index results is shown in Table 2.

**Table 2:** Evaluation criteria according to the index result

Index		Values	Comprehensive assessment
Contamination factor (CF)	1	CF < 1	Low contamination factor
	2	1 < CF ≤ 3	Moderate contamination factor
	3	3 < CF ≤ 6	Considerable contamination factor
	4	CF > 6	Very high contamination factor
Ecological risk index (RI)	1	RI ≤ 150	Low ecological risk
	2	150 < RI ≤ 300	Moderate ecological risk
	3	300 < RI ≤ 600	High ecological risk
	4	RI > 600	Significantly high ecological risk
Pollution load index (PLI)	1	PLI ≤ 1	Uncontaminated by meals
	2	PLI > 1	Contaminated by metals

### Statistical analysis

#### Correlation analysis and Principal component analysis

Statistical calculations were performed using IMB SPSS 23. The data were checked for normal distribution (Shapiro–Wilk’s test). Based on the statistical verification, the normality was found to be significant (P < 0.05). Log transformation was used to decrease the variability of data and make data close to the normal distribution. Because in all the cases, the obtained data were not normally distributed, Spearman method was applied for determining the correlations of the metal(loid)s.

Principal component analysis (PCA) was applied to identify the different groups of metal(loid)s that were correlated, and therefore, it showed similar behaviors and possible common origin of the elements. PCA reduces the size of the data set, explaining the correlation between variables through a small group of principal components, without losing too much information (Vega et al., 1998). This study used the Kaiser criterion to reduce the number of variables and correlation matrix by applying a varimax rotation. The criterion objective was to discard components that were not informative and keep the ones that contain most of the information from the initial variables, therefore only those factors with eigenvalues greater than 1 were retained.

#### 2.4.2 Hierarchical cluster analysis

Statistical computations were performed using hierarchical cluster analysis (Ward’s method). The method used the squared Euclidean distance as a similarity measure. Cluster analysis was dividing the metals into classes, as a result, similar metals behavior/origin were in the same cluster. In fact, the groups were not known prior to apply this mathematical analysis and no assumption was made about the distribution of the variables (Stahl & Sallis, 2012). The degree of the association was determined by the closer distance between metal(loid)s (Kent & Vujakovic, 2009).

#### Mann–Whitney U test

Mann*–*Whitney was used to evaluate whether there exists any significant difference only between two riverbeds. The null hypothesis stated that there is no significant difference in the metal(loid)s mean concentration. Accordingly, an alternative hypothesis argued that these differences exist.

## Results and discussion

### Physic-chemical properties

**Fig. 2:**
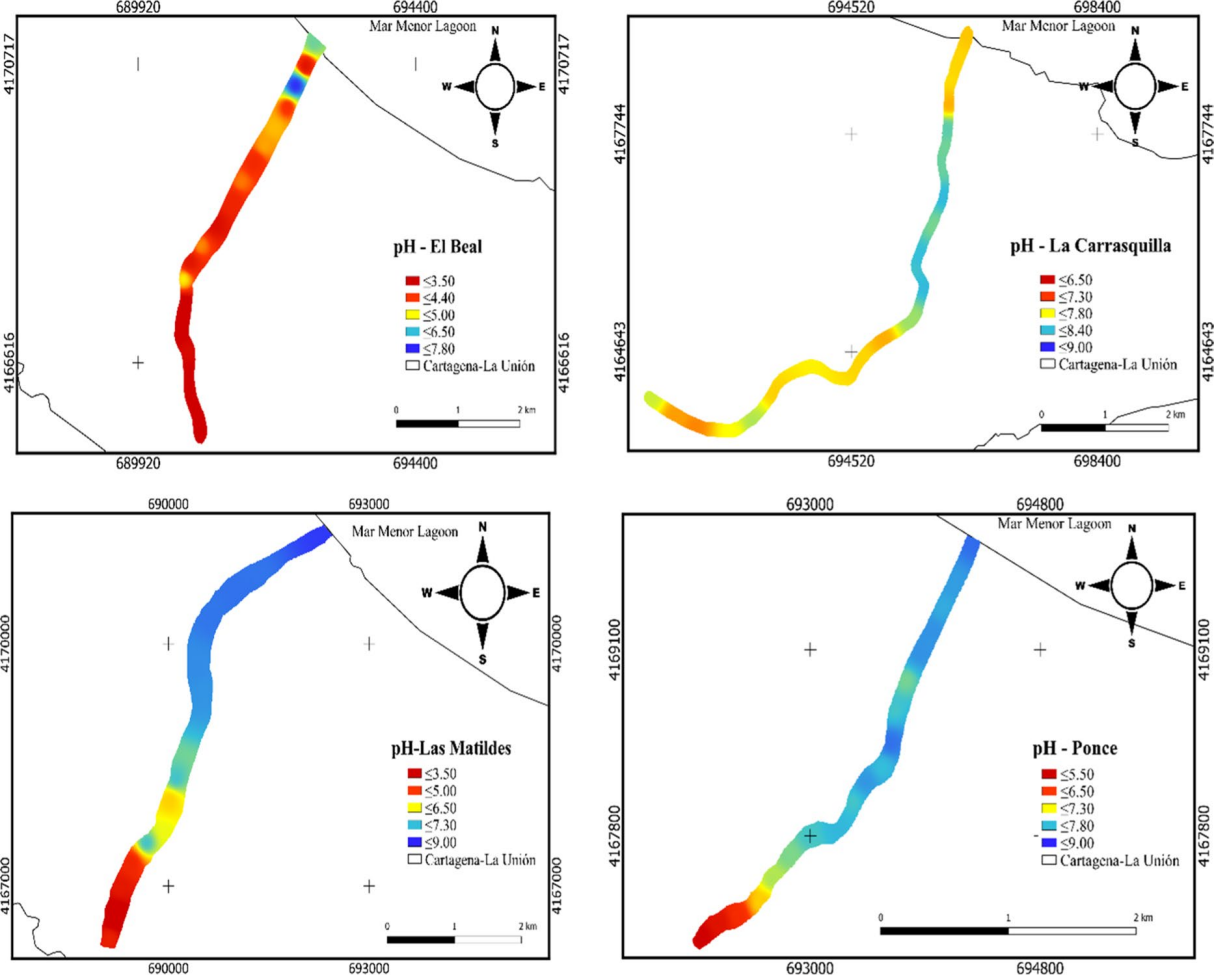
pH spatial distribution in soil samples from dry riverbeds

The pH values along the four dry riverbeds studied ranged between ultra-acid 2.1 (EB) in the headwater to strongly alkaline 8.76 (LM) near the coast (Fig. 2) (Soil Survey Division Staff, 1993). In general, these values tended to decrease as they moved away from the headwater until approached the coast of the Mar Menor lagoon, the acid mine drainage (AMD) from the headwaters and the mine waste dragged by the rainwater were responsible for it. The inorganic carbon content increased as it moves away from the headwaters, reaching values from 0.74% (EB), to 7.52% (LC) (supplementary material). In the case of EB and PN, the organic carbon content increased at points not far from the headwater zone (supplementary material) associated with the spontaneous growth of vegetation in uncontaminated areas. The differences in organic matter content along the riverbeds were due to soil disruptions caused by mine wastes (Mccauley et al., 2017), which prevents plant colonization (Chrastný et al., 2012). On the other hand, the textural analysis classified the sediments as loamy sand in the four riverbeds. The highest percentages of clay and silt were found near the coast while sand percentages were generally homogeneous along the riverbeds. Widely varying electrical conductivity (EC) values were observed at samples located near the headwaters (supplementary material), which values ranged from very slightly saline, 2.71 mS cm^−1^ (EB), to moderately saline, 9.89 mS cm^−1^ (LC) (Soil Survey Division Staff, 1993). This is most likely due to the contribution of materials rich in metallic sulfides from the dragging of wastes in mining headwaters. Sulfate (SO_4_^−2^) was the most dominant anion, the mean concentration in EB, was 10,419 mg kg^−1^, and the lowest value was found near the headwater. Meanwhile, the highest concentration of SO_4_^−2^ in LC (1461 mg kg^−1^) was found close to the headwater. Mine wastes containing high sulfide concentrations are the main source of salts, especially sulfates contributing to environmental pollution (Candeias et al., 2014). The cause is due to the potential production of acid mine drainage formed by the oxidation of sulfide minerals, commonly pyrite (Garcia-Lorenzo et al*.,* 2016). In addition, the dominant cation was calcium (Ca^+2^) with mean concentrations in LC was 1277 mg kg^−1^ and PN was 624 mg kg^−1^ (supplementary material).

### Concentration of metal(loid)s

Statistical results of the total metals and arsenic concentrations are summarized in Table 3. This table shows the maximum, minimum and means concentrations for the four dry riverbeds studied in the Cartagena-La Unión mining area. Considerable variations in metal(loid)s concentrations between different segments of the riverbeds were observed. According to the results, the mean concentration of metal(loid)s between riverbeds was significatively different (*p* > 0.5) (As, Cr, Cd, Cu, Fe, Ni and Mn), LM showed a Pb (9690 mg kg^−1^) concentration at least 3.35 higher than LC and PN. However, Zn concentrations were no significant differences between the riverbeds with a mean concentration of 4081 mg kg^−1^ (Table 3). In addition, the results for Pb showed that there were no significant differences between LC (2896 mg kg^−1^) and PN (2842 mg kg^−1^). Garcia-Garcia (2004) reported 3500 mg kg^−1^ Zn in LC near the coast. Martín-Crespo et al. (2020) reported the following concentration range in LM: As (42 to147 mg kg^−1^), Cd (3.2 to 11.9 mg kg^−1^), Pb (2450 to 6770 mg kg^−1^), and Zn (994 to 4680 mg kg^−1^).

**Table 3:** Summary statistics of total metal concentrations in soil samples (mg kg^−1^)

	El Beal	La Carrasquilla	Las Matildes	Ponce
As				
Max	448	409	643	217
Min	205	6.56	41.5	6.32
Mean	338a	123bd	243c	69.2d
Cd				
Max	26.8	32.5	29.9	51.7
Min	3.17	0.99	1.56	1.63
Mean	9.15a	12.8ab	15.5ab	20.0b
Cr				
Max	34.1	20.3	19.7	23.0
Min	17.4	7.68	11.0	8.87
Mean	24.7a	14.6b.cd	16.5 cd	15.9d
Cu				
Max	128	7.50	105	86.1
Min	47.4	10.5	13.5	7.58
Mean	85.7a	37.7bd	67.8c	39.3d
Fe				
Max	117,898	142,596	155,685	72,679
Min	78,626	11,231	8790	6061
Mean	100037a	57653bcd	87629c	28329d
Mn				
Max	5898	8723	6978	5570
Min	822	921	169	703
Mean	2287a	3793ab	4437b	3037a
Ni				
Max	21.4	22.8	17.0	24.7
Min	7.21	6.81	3.64	7.46
Mean	13.8a	17.7b	13.0a	16.4ab
Pb				
Max	9459	10,137	21,540	6231
Min	1204	286	1715	373
Mean	4344a	2896b	9690c	2842a.b
Zn				
Max	7311	9590	6603	15,858
Min	1767	263	1142	411
Mean	3358a	3540a	3857a	5570a

Different letters indicate significant differences (*p* < 0.05) between each metal(loid)s concentration analyzed by Mann–Whitney U test

**Fig. 3:**
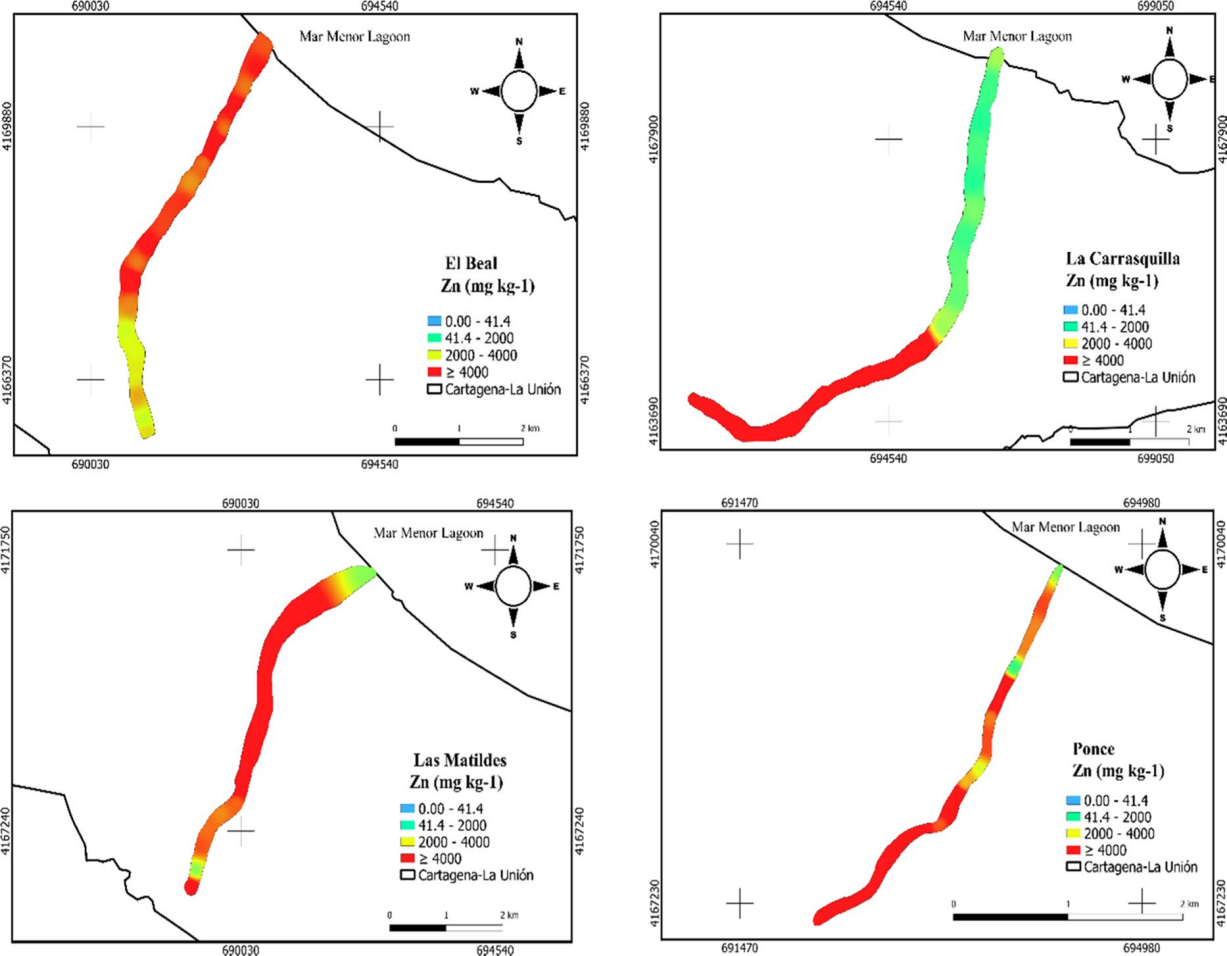
Zinc (Zn) spatial distribution in soil samples from dry riverbeds

The spatial distribution of the 9 metal(loid)s analyzed presented variations along the surface (supplementary material). However, the four dry riverbeds showed a common behavior with As maximum concentrations close to the headwater in EB, (448 mg kg^−1^), LC (409 mg kg^−1^), LM (643 mg kg^−1^) and PN (217 mg kg^−1^) (supplementary material). EB (26.8 mg kg^−1^) and LM (29.9 mg kg^−1^) had maximum Cd concentration near the coast, while LC and PN exhibited it close to the headwater in a segment characterized by acid pH (supplementary material). EB, LM and PN presented Pb concentrations at least 100 times higher than the background reference values (Martínez-Sánchez et al., 2007) in almost all the sample points. In general, EB, LC, LM and PN presented concentration over the background values for As, Cd, Cu, Mn, Ni, Pb, and Zn. García et al. (2003) reported Pb concentration in EB over 19,100 mg kg^−1^, with the maximum values close to the headwater. On the other hand, as is shown in (Fig. 3) EB, LM and PN on most of the sample points presented Zn concentration at least 100 higher than the background values.

### Identification of metal(loid)s contamination

#### Contamination factor

**Fig. 4:**
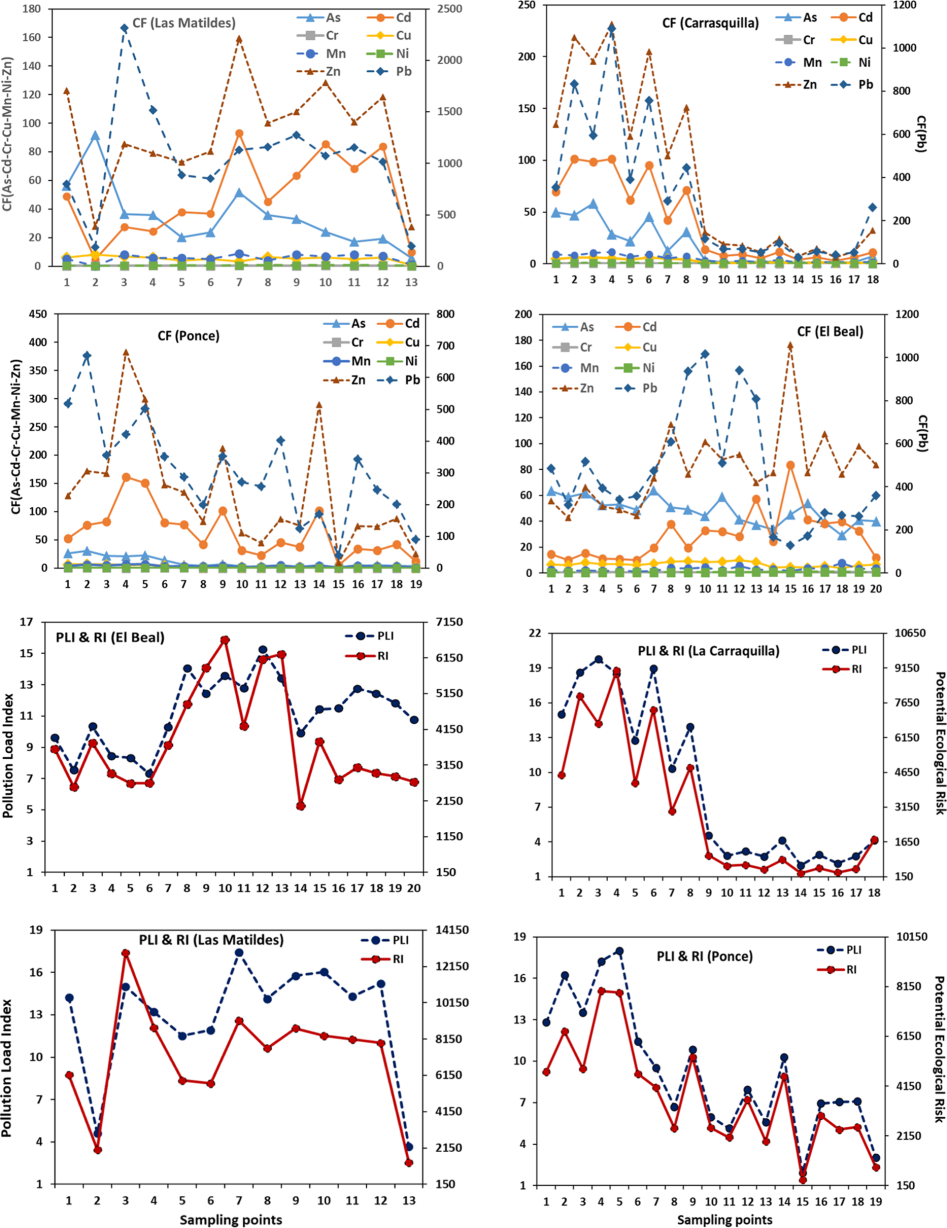
Contamination factor, pollution load and ecological risk indices in the four dry riverbeds

The contamination factor (Cf) revealed a very high contamination in the four riverbeds (Cf > 6) (Hakanson, 1980). Overall, results showed As, Cd, Pb and Zn with the highest contamination factor (Fig. 4). The two highest mean contamination factors in the four riverbeds were Pb with a mean value of 530 and Zn with a 98. The mean concentration of Pb and Zn in the four riverbeds were at least 6.30 times higher than the reference background values (Martínez-Sánchez et al., 2007), with samples that reached over 1500 times the geochemical background, which indicates that there is anthropogenic contamination, where Pb concentration in LM was higher than the content reported by Conesa et al. (2008) (Pb = 7000 mg kg^−1^). In all riverbeds the highest Cfs were Pb > Zn > Cd > As, except EB, where As was higher than Cd. The maximum Cfs values in most of the points were located in areas near the headwaters, except in EB. However, in the other three dry riverbeds, the concentration levels in the intermediate points showed peaks, which could be attributed to the accumulation of contaminated waste in these points. The mean Cf value for Cu ranged between considerable (3 < Cf < 6) in LC, LM and PN, to very high in EB (Cf > 6). In the cases of Cr and Ni in the four dry riverbeds, the mean Cfs values were lower than 1.

#### Pollution load index

The pollution load index introduced by Tomlinson et al. (1980) was used to study the impact of all metals at the same time, since values of PLI > 1 classified soil as contaminated by metals. The index was calculated for the 4 dry riverbeds and the 8 metal(loid)s analyzed (As, Cd, Cr, Cu, Mn, Ni, Pb, and Zn). According to the PLI, Pb and Zn were the elements that contributed most to the PLI levels. Results showed that mean PLI values were estimated between 8.8 in LC and 15.2 in LM (Fig. 4). The PLI in LC and PN tended to decrease as the sampling points move away from the headwater area, which can be attributed to direct discharges from other anthropogenic sources. Values of PCI for LC ranged from 19.8 close to the headwater to 2.76 near the coast, while PLI for PN ranged from 16.2 to 6.94. In contrast, the PLI in EB and LM had a similar behavior along both riverbeds (Fig. 4). Contamination along other riverbeds had been reported by other authors (Pavetti et al., 2006), which reported that despite mining wastes were transported along the riverbed, the highest metals concentrations were found in the headwaters. In addition, Pavetti et al. (2006) reported metals concentrations (Cu, Pb and Zn) from El Gorguel, close to the headwater of 1691 mg kg^−1^ for Cu, 18,102 mg kg^−1^ for Pb, and 10,913 mg kg^−1^ for Zn, while on sample points in the mouth the concentrations were 1050 mg kg^−1^ for Cu, 1949 mg kg^−1^ for Pb, and 6747 mg kg^−1^ for Zn.

#### Ecological risk index

The ecological risk index (RI) had similar behavior to the pollutant load index (PLI). All points in EB, LC and LM were classified with a high ecological risk RI > 600. The mean value of the RI in PN was 3798 and only one sampling point (Nº 15) had a RI < 600, even indicating a high ecological risk. Results revealed that Cd and Pb had the highest impact in the values of the ecological risk index. The contribution for Pb as an individual element was 72.9% in LM, 61.5% in EB, 51.2% in LC and 41.7% in PN. Generally, high ecological risk grades were identified at the sample locations close to the headwaters. Gonzalez-Fernandez et al., (2011a, 2011b) concluded that the content of Pb, Zn, Cu, and As at EB riverbed should be considered a highly polluted area and imply an important environmental risk, reporting total concentration of Pb ranges from 3100 to 62,500 mg kg^−1^, Zn range between 3000 and 38,500 mg kg^−1^, As 100 and 1600 mg kg^−1^, and Cu around 500 mg kg^−1^.

### Source of metals and arsenic

#### Correlation analysis and Principal component analysis (PCA)

In order to more accurately identify and interpret the source of the metal(loid)s, correlation analysis was combined with principal component analysis, summarized in Table 4. Results from PCA in EB showed three principal components, which explained 82.9% of the total variance. PC1 was responsible for 43.6% of the total variance and was dominated by Cd and Zn, with high loadings of 0.89 and 0.88, respectively, whose presence is mainly associated with sphalerite (ZnS) from mining operations. Sphalerite (ZnS) is the primary geologic source of Zn and Cd around the world (Alloway, 2012) and in the study area. Meanwhile, PC2 explained 22% of the total variance and was loaded on Cr, Ni, Mn and Fe, representing a lithogenic origin from weathering and erosion of parent material. PC3 was represented by Cu, As and Pb, which accounted for 17.3% of the total variance. In this case, Cu had the highest loadings on PC3 with 0.94 and it was associated with metals impurities from primary minerals (Manteca & Ovejero, 1992), being related to mineable ores as galena (PbS).

**Table 4:** Results of principal component analysis **(**PCA) for metal(loid)s in the soils from riverbeds

Rotated loading matrix										
Meta(loid)s	Beal	Carrasquilla				Matildes			Ponce	
	Components									
	PC1	PC2	PC3	PC1	PC2	PC1	PC2	PC3	PC1	PC2
As	− 0.57	− 0.51	0.22	0.88	0.35	− 0.22	0.95	− 0.11	0.95	0.24
Cd	0.89	0.06	0.05	0.95	0.30	0.97	− 0.01	− 0.04	0.41	0.84
Cr	0.48	0.62	− 0.46	0.38	0.89	0.71	− 0.07	0.54	0.41	0.72
Cu	− 0.07	− 0.09	0.94	0.91	0.36	− 0.14	0.71	0.54	0.94	0.31
Fe	− 0.46	0.67	0.22	0.92	0.37	0.23	0.94	− 0.10	0.93	0.32
Mn	0.16	0.95	0.11	0.93	0.32	0.79	− 0.11	0.47	0.61	0.69
Ni	0.62	0.63	− 0.42	0.17	0.94	0.91	− 0.21	0.32	0.04	0.96
Pb	− 0.01	0.14	0.96	0.94	0.10	0.32	− 0.04	0.88	0.87	0.28
Zn	0.88	0.03	0.05	0.96	0.25	0.96	0.17	0.11	0.36	0.87
Variance%	43.6	22.0	17.3	81.4	13.2	51.8	26.3	12.2	71.8	16.6

In LC riverbed, PCA identified two components that explained about 94.6% of the total variance. The first PC accounted for 81.4% and grouped As, Cd, Cu, Fe, Mn, Pb, and Zn. The significant correlation coefficients between metals could result from the same sources (Li & Feng, 2012), also a similar behavior could indicate that these metals have been extracted and handled with the same process. There were positive strong and significant correlations between As, Cd, Cu, Fe, Mn, Pb, and Zn. The main stronger correlations were found between Pb–Zn (*r* = 0.99), Cd-Zn (*r* = 0.99) and Fe–Mn (*r* = 0.95). A strong and significant correlation was also reported by Fernández-Naranjo et al. (2020), who found high correlation of Cd-Zn in tailings from the area (*r* = 0.97). These results reflected a common high concentration near the headwater which suggests anthropogenic impact related to the sulfur minerals. The second PC accounting for 13.2% of the total variance, and it was correlated with Cr and Ni, both with concentration under the background levels, indicating that these elements were derived from lithogenic sources. In LM riverbed, PCA extracted 3 PCs, which accounted for about 90.3% of the total variance. Based on the loading distribution of the element, Cd, Cr, Mn, Ni and Zn constituted the PC1, with 51.8% of the total variance, where Cd and Zn had the highest loading with 0.97 and 0.96 values, suggesting mine origin from sphalerite (ZnS), while Cr, Mn and Ni concentration suggested being impurities of this mineral. PC2 was composed of As, Cu and Fe explained the 26.3% of the total variance, likely with an origin in the pyrite mined in this district. The third component (PC3) accounted for 12.2% with high loading only on Pb, associated with the galena (PbS). In addition, correlation analysis showed that Pb, had no significant correlation with almost any element, except with Mn (*r* = 0.69), suggesting that the anthropogenic input for this element was relatively high. Gonzalez-Fernandez et al., (2011a, 2011b) reported that Pb-enriched levels are due to the absence of secondary transport processes of Pb due to the low solubility of PbSO_4_. These increases are probably caused by the more important mining periods in Cartagena–La Unión mining district (Manteca Martinez et al., 2005). During the Roman ages, Pb production reaches a peak of production up to 45,000 Tm year^−1^ (Moreno-Grau et al., 2002).

PN riverbed had 2 principal components, the first accounted the 71.8% of the total variance, which was positively dominated by As, Cu, Fe and Pb, suggesting mine wastes as a source of these metal(loid)s, where Fe and Pb loading were not as high as As and Cu. Additionally, PC1 presented negative correlations with pH, Gonzalez-Fernandez et al., (2011a, 2011b) reported the distribution of Pb, Cu, and As contents inversely related to the pH values. Conesa et al. (2008) investigated mine tailings from the mining district Cartagena-La Unión and reported that acidic tailings had 5 times more As and 4.5 times more Cu. The PC2 explained 16.6% of the total variance and was high loading by Cd, Cr, Mn, Ni and Zn, suggesting mine origin from sphalerite (ZnS) for Zn, while Cd, Cr, Mn and Ni being impurities of this mineral. Gabarron et al. (2018) studied metal(loid)s concentrations in the mining district Cartagena- La Union indicated that Cr and Ni were statistically higher in natural/agricultural soils than in mining waste, suggesting a geological origin of this metal.

#### Cluster analysis (CA)

**Fig. 5:**
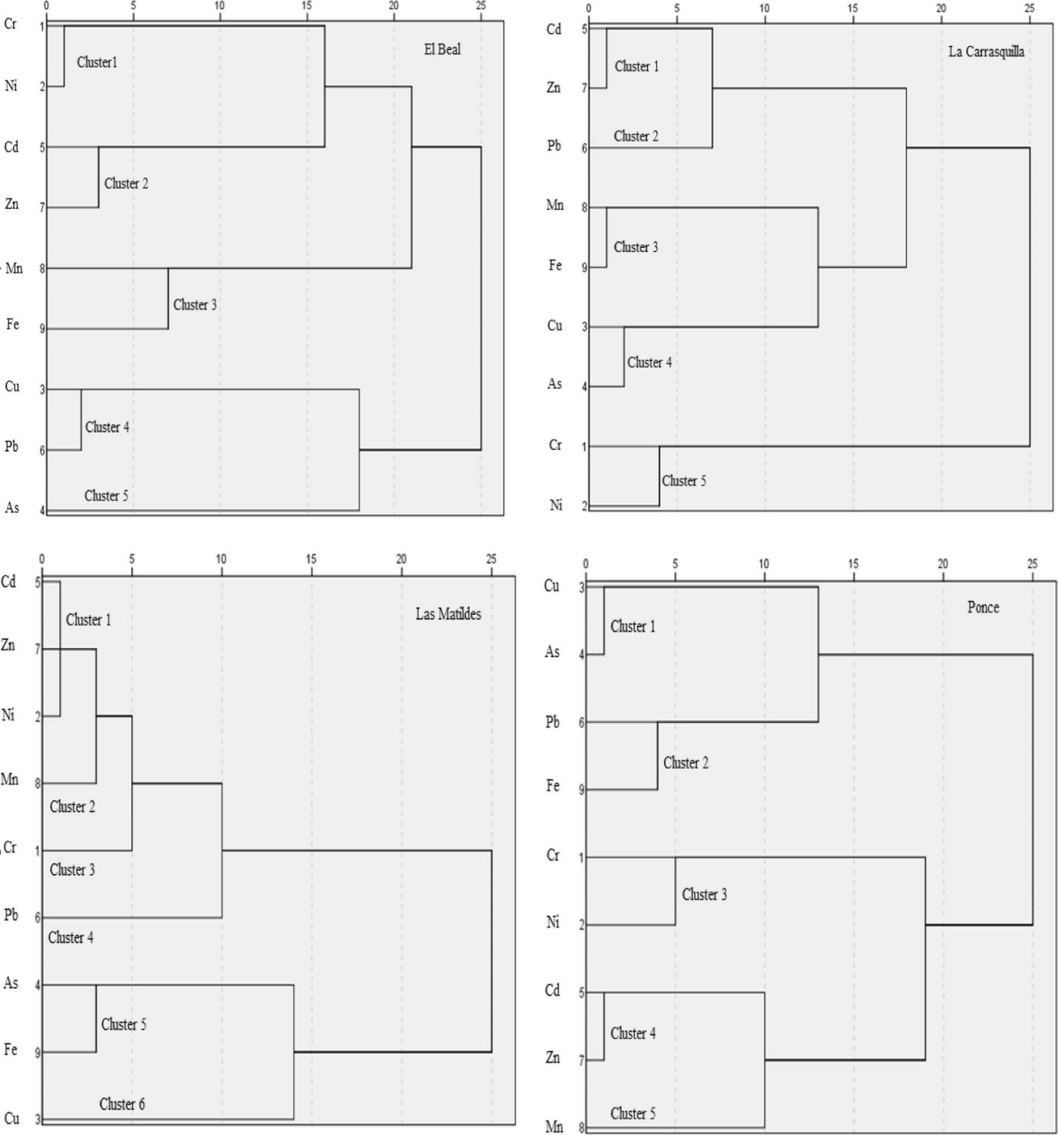
Dendrogram obtained by cluster analysis for metal(loid)s contents in soil samples from the riverbeds

Hierarchical cluster analysis was also carried out to identify the possible sources. The results were illustrated in a hierarchical dendrogram (Fig. 5). The classifications for these dendrograms were similar to the correlation analysis and the PCA analysis. Based on 9 metal(loid)s (As, Cd, Cr, Cu, Fe, Mn, Ni, Pb and Zn) concentrations, metal(loid)s from riverbeds were divided into clusters. EB riverbed contains 5 clusters, the second cluster was characterized by the high concentrations of Cd and Zn, which is corroborated by PC1, both persisted along the riverbed, even near the coastal area. The relationship between Zn and Cd in soils and sediments—within the sulfide mining zones-are commonly observed (Rodriguez et al., 2009). The reasons are due to the chemical similarity of Zn and Cd (e.g., electron configuration, electronic charges, and electronegativity), dissolution from associated secondary-mineral phases commonly displays similar geochemical behavior in mine-waste environments (Blackmore et al., 2018). On the other hand, clusters 1 and 3 were included in the same group due to its same behavior on Cr, Ni, Fe and Mn concentrations. Finally, clusters 4 and 5 including Pb, Cu and As, which were related to anthropogenic origin.

Five clusters could be distinguished in the dendrogram from LC riverbed. Clusters 1, 2, 3, and 4 belonged to a group with high concentrations of As, Cd, Cu, Fe, Mn, Pb and Zn located close to the headwater, associated with PC1, which suggest an anthropogenic origin. The common behavior from some of those metal(loid)s could be the result of improper management, which results in the oxidation of sulfide minerals. Metalliferous acidic mine drainage leads to the leaching of concentrated metallic ions like Mn, Fe Cu, Pb and Zn (Vega et al., 2006). In contrast, Cr and Ni showed a homogenous concentration along the riverbed, being grouped in cluster 5, and according to the level of concentration, suggested a natural origin of these metals in this riverbed.

LM riverbed, HCA categorized the 9 metal(loid)s into six clusters, a group included clusters 1, 2, 3, and 4, and accounted for the concentration with a homogenous behavior along the riverbed. Clusters 1, 2 and 3 were corroborated by PC1, suggesting mine origin from sphalerite of the metals included in this cluster, while cluster 4 is related to galena. The second group accounted for clusters 5 and 6, which had the highest concentrations of As, Cu, and Fe, close to the headwater, which origin likely is the pyrite. Arsenic is associated with different types of mineral deposits, and especially has a strong affinity with sulfide ores (Lazo et al., 2007). In fact, As is a natural component of Pb, Zn, and Cu, ores and consequently may contaminate soils, and sediments, during mining and smelting operations (Garelick et al., 2009).

The HCA analysis showed in PN riverbed that metal(loid)s were clustered into two main groups. One of them included clusters 1 and 2, which accounted the highest concentrations of As, Cu, Fe, and Pb and showed high contamination in the headwater. Cluster 2 represented that this mining area, Sierra Minera of Cartagena, was rich in iron–manganese mineralized veins. In the past, this huge iron–manganese ore occurrence led to intensive mining activity for its industrial benefits, which was in great demand for steel foundries (Martínez-Pagán et al., 2013). Meanwhile, PC2 represent cluster 3,4 and 5, where Cd-Zn-Mn and Ni–Cr present a strong correlation. According to Martínez-Martínez (2009), the concentrations of Ni and Cr in the studied mining waste, in no case it exceed the generic reference levels, therefore, there are no indications of Ni and Cr contamination in the study area because of mining activity.

## Conclusions

Concentrations of As, Cd, Cu, Pb, Mn and Zn for all riverbeds were higher than the background values, suggesting anthropogenic sources of these metals, in addition, the pH and salinity were affected by mining activity, where the highest acidity and salinity was found close to the headwater of the riverbeds. High sulfate (SO_4_^−2^) concentration was the main anion increasing the soil salinity in the riverbeds, which could come from acid drainage formed as a result of the oxidation of sulfide minerals. La carrasquilla (LC), Las Matildes (LM), and Ponce (PN) presented the highest concentration of Pb and Zn near the headwater, decreasing as they approached the coast along the riverbed. Meanwhile, El Beal (EB) showed the highest concertation of Pb and Zn between points 9 and 13, showing an accumulation of mine waste in this area. In contrast, As presented the highest concentrations close to the headwater on the four dry riverbeds. El Beal (EB) and Las Matildes (LM) showed maximum concentrations of Cd in the middle of the riverbed, while La Carrasquilla (LC) and Ponce (PN) were in the headwater.

Based on the indices used to evaluate the pollution degree (Cf, PLI, and RI), Pb and Zn showed the highest degrees of contamination in the four dry riverbeds. The same two elements contributed the highest values to the PLI index. In La Carrasquilla (LC) and Ponce (PN), the PLI index decreased while moving away from the headwaters, however, El Beal (EB) and Ponce (PN) presented similar values along the riverbeds. Those behaviors were confirmed by RI index, however, in this case, Cd and Pb were the two main contributors, both associated with mine activities.

The correlation analysis showed a strong and significant correlation between Cd and Zn in the four dry riverbeds which suggested the same source, sphalerite (ZnS). PCA analysis established that the sources of metal(loid)s in sediments of the four dry riverbeds differed: (1) In El Beal (EB), the lithogenic origin was the main source of Cr, Ni, Fe and Mn and anthropogenic input accounted for As, Cd, Cu, Pb, and Zn. (2) In the La Carrasquillas (LC), Las Matildes (LM) and Ponce (PN), the lithogenic origin accounted for Cr and Ni, while anthropogenic input for: As, Cd, Cu, Fe, Mn, Pb and Zn. Therefore, it can be concluded that these four dry riverbeds should be considered highly contaminated by Cd, Cu, Pb and Zn, prospective human risk and ecological assessment study is needed to estimate the effects.

### Supplementary Information

Below is the link to the electronic supplementary material.

Supplementary file1 (DOCX 3568 KB)

## Data Availability

The authors confirm that the data supporting the findings of this study are available within the article and its supplementary materials**.**

## Declarations

### Human and animal research

The authors confirm that this study does not involved animals, their data or biological material.
