# Supplementary material for: Spatial distribution and pollution evaluation in dry riverbeds affected by mine tailings
Source: Environ Geochem Health. 2023 Jan 16;45(12):9157–73. doi: 10.1007/s10653-022-01469-5 (PMC10673978; doi:10.1007/s10653-022-01469-5)
Supplement: Supplementary file 1 — Supplementary file1 (DOCX 3568 KB) [file 10653_2022_1469_MOESM1_ESM.docx]

# Spatial distribution and pollution evaluation in dry riverbeds affected by mine tailings

**Environmental Geochemistry and Health**

# Cuevas J.G.*, Faz A., Martínez-Martínez S., Gabarrón M., Beltrá J.C., Martínez J., Acosta J.A.

*Sustainable Use, Management and Reclamation of Soil and Water Research Group, Universidad Politécnica de Cartagena, Paseo Alfonso XIII 48, 30203, Cartagena, Spain. *corresponding author*

[**Jose.cuevas@upct.es**](mailto:Jose.cuevas@upct.es)

**Supplementary Material**

| 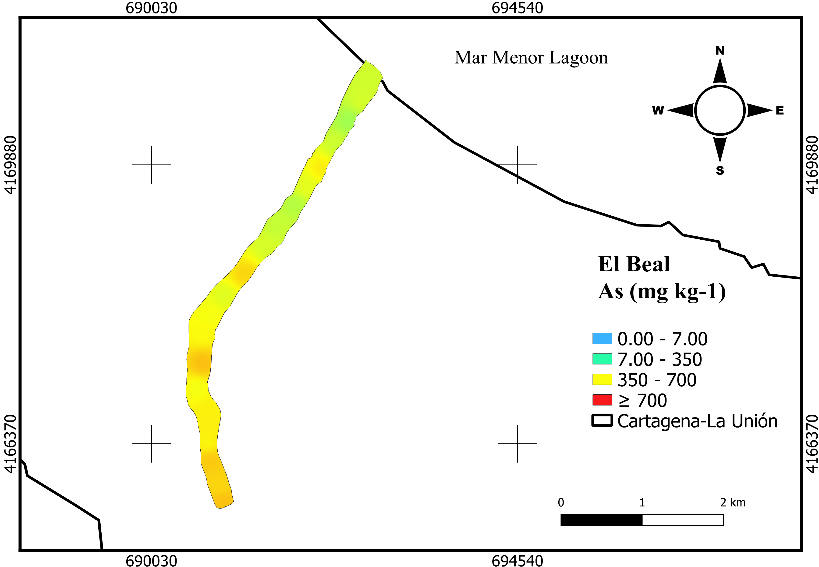 | 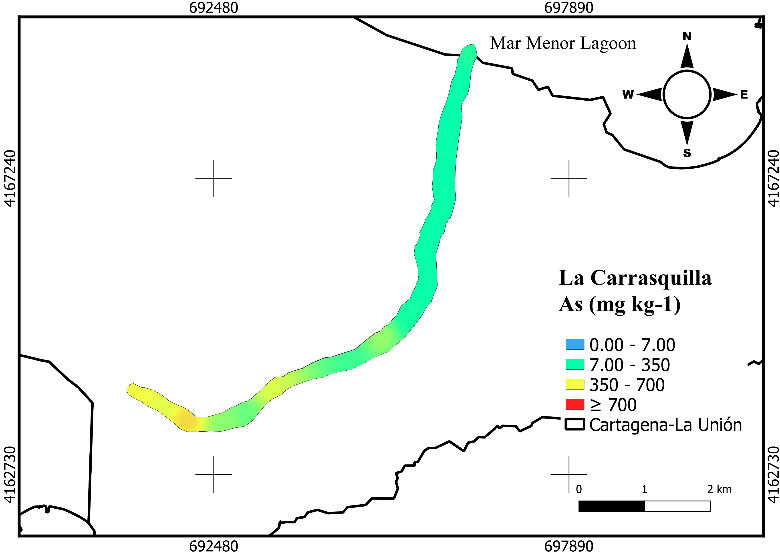 |
| --- | --- |
| 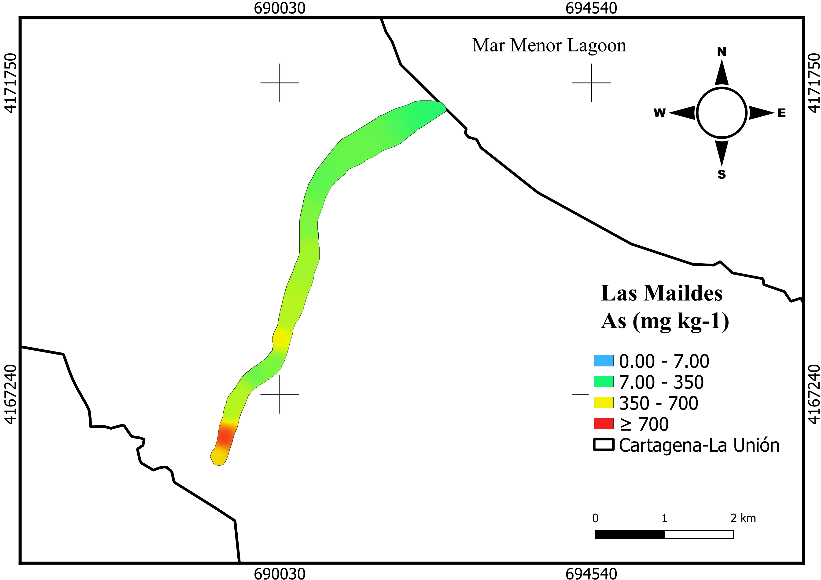 | 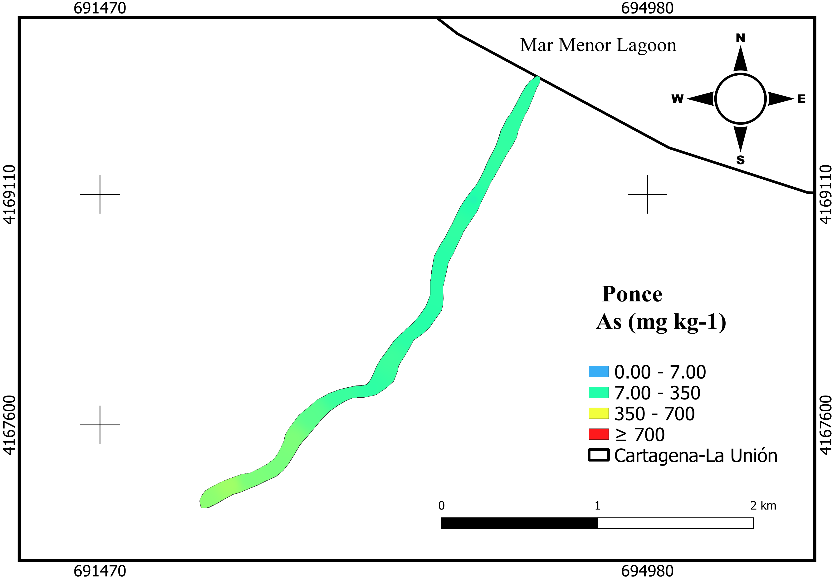 |

**Fig.1** As spatial distribution in soil samples from dry riverbeds.

| 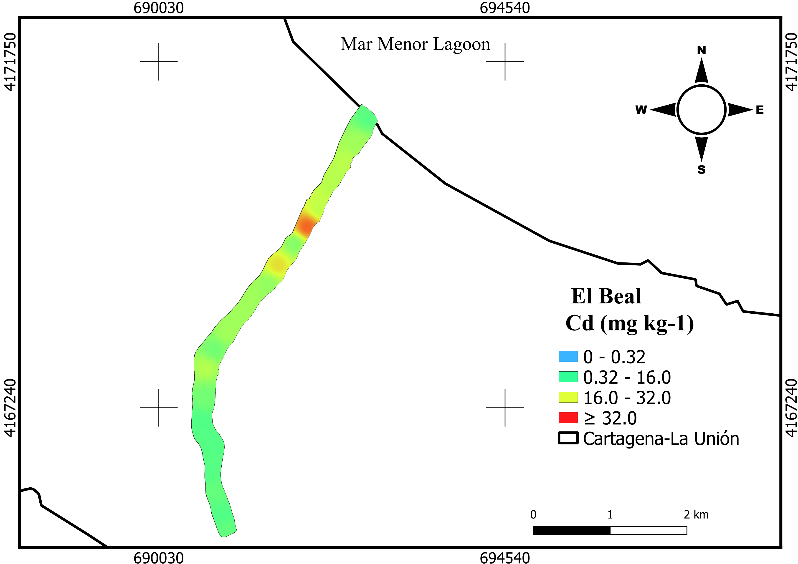 | 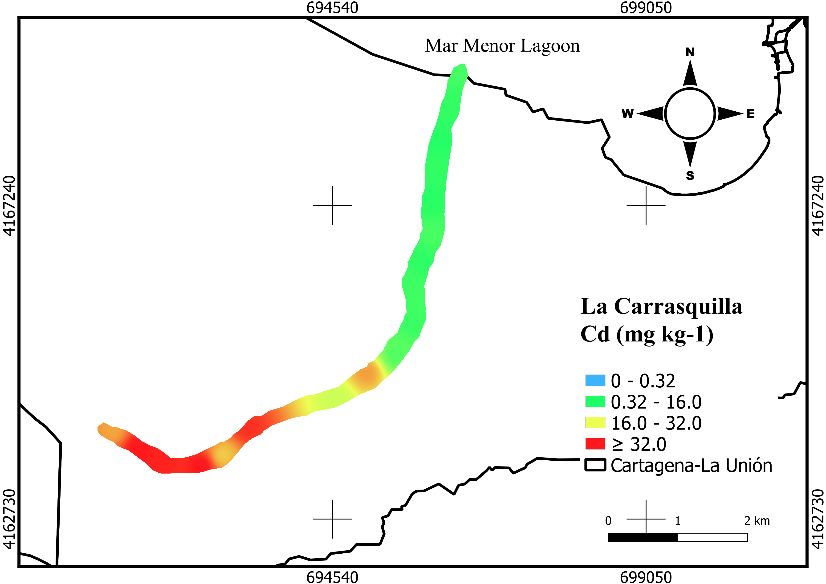 |
| --- | --- |
| 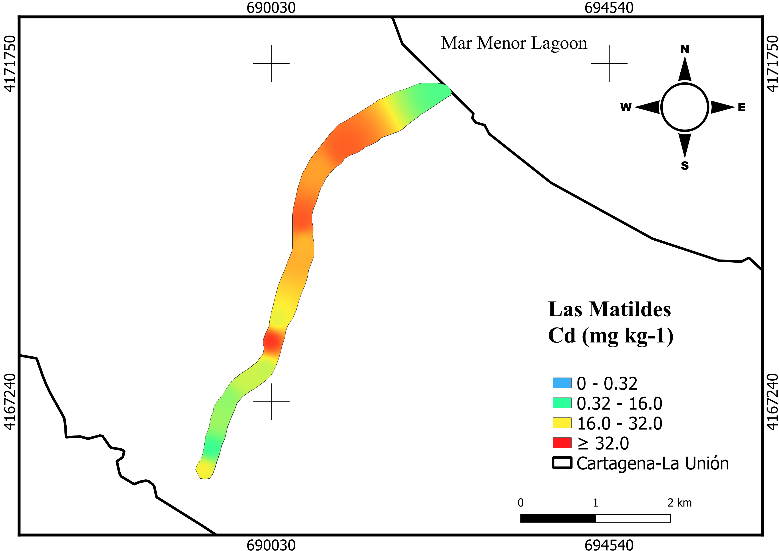 | 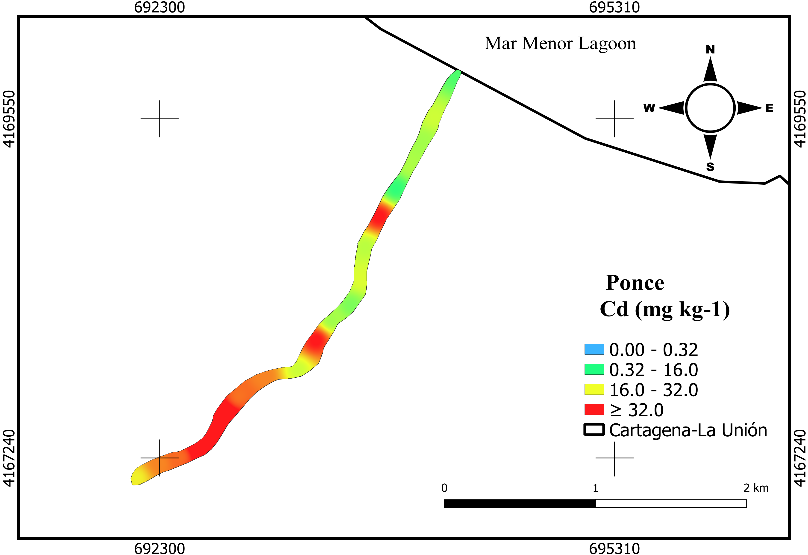 |

**Fig.2** Cd spatial distribution in soil samples from dry riverbeds.

| 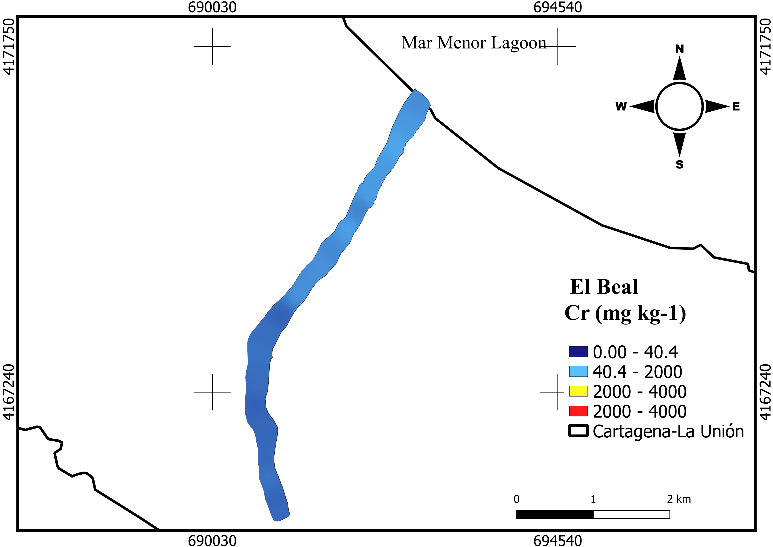 | 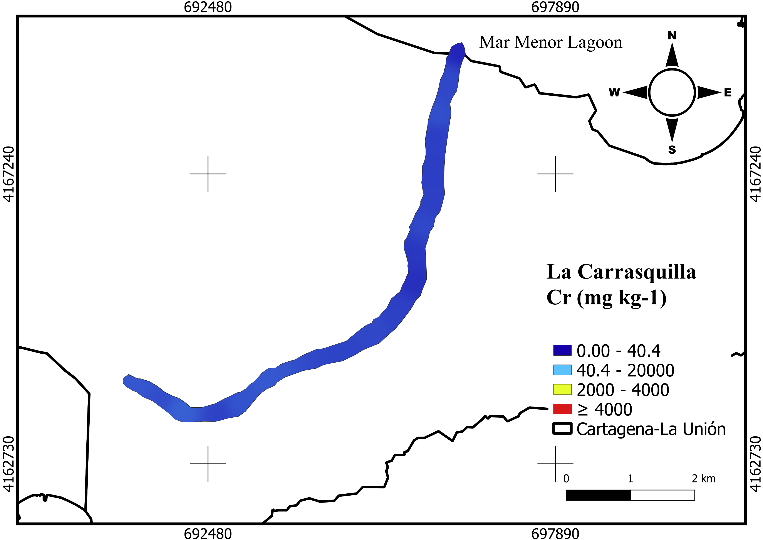 |
| --- | --- |
| 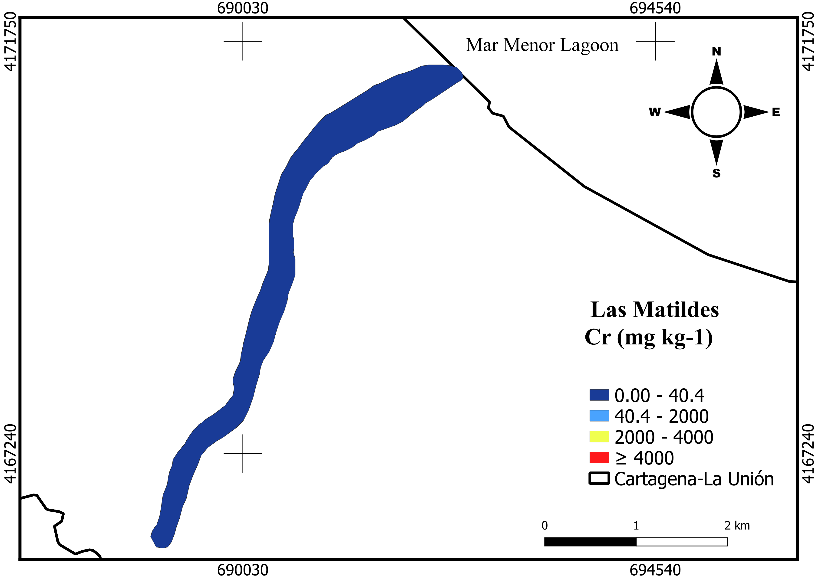 | 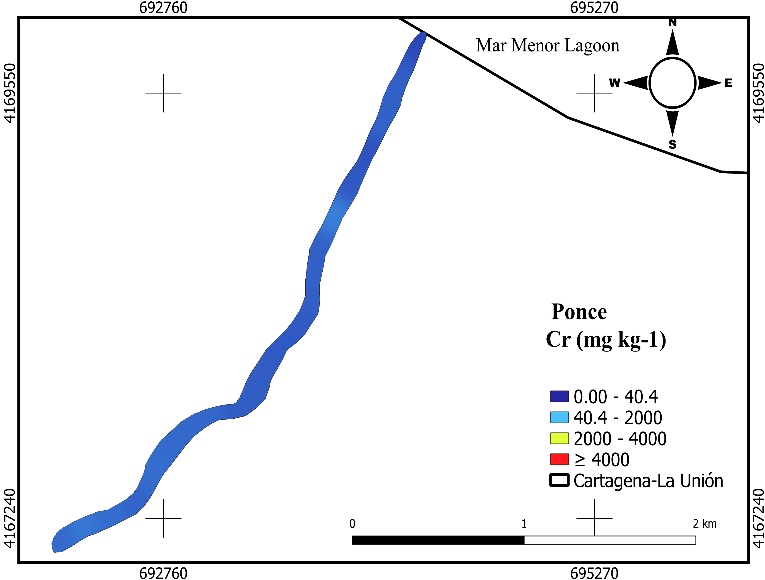 |

**Fig.3** Cr spatial distribution in soil samples from dry riverbeds.

| 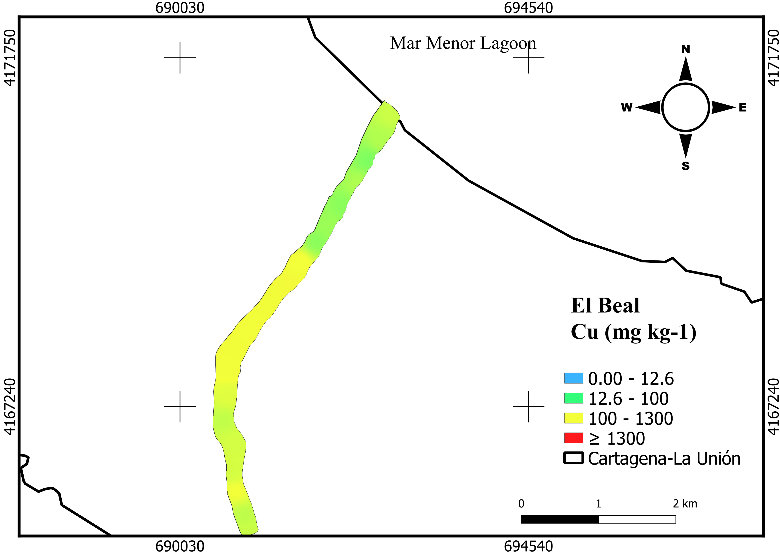 | 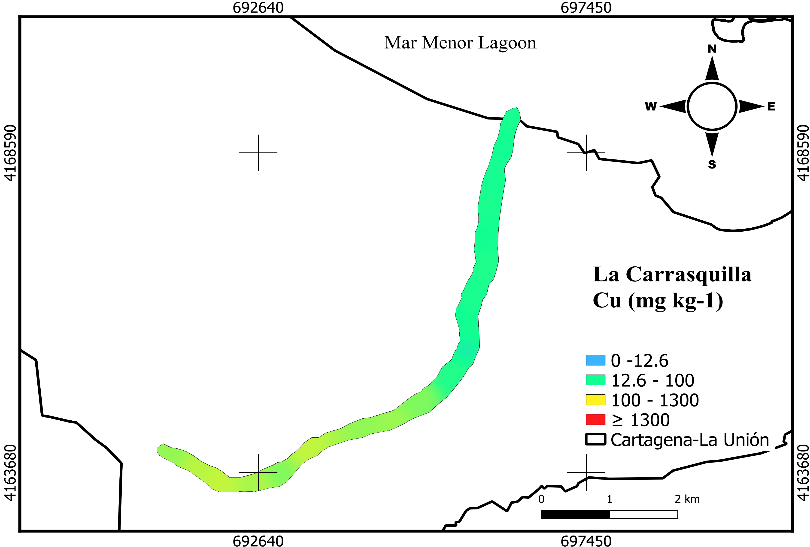 |
| --- | --- |
| 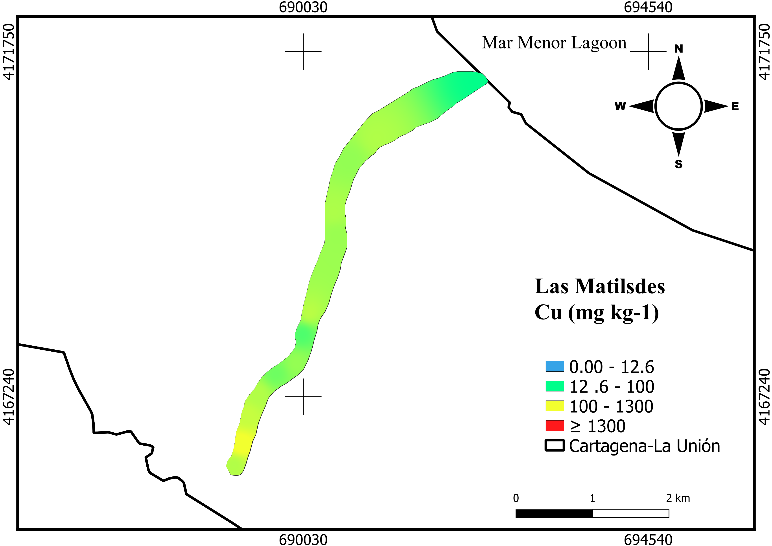 | 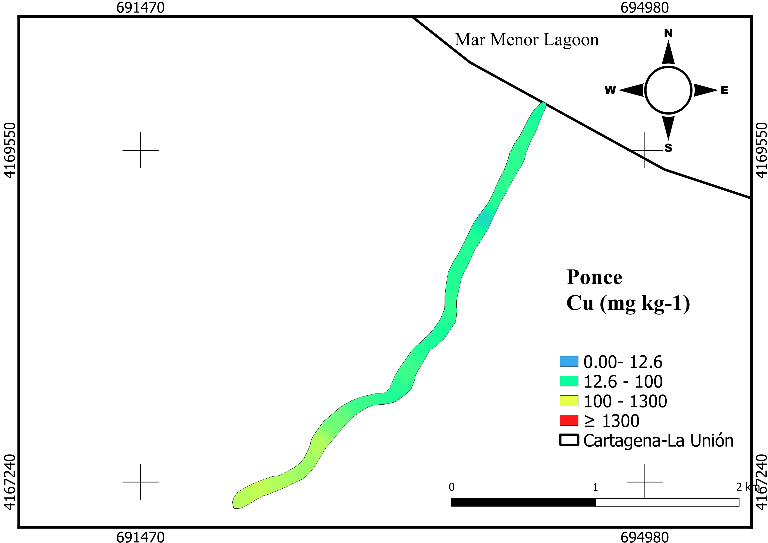 |

**Fig.4** Cu spatial distribution in soil samples from dry riverbeds.

| 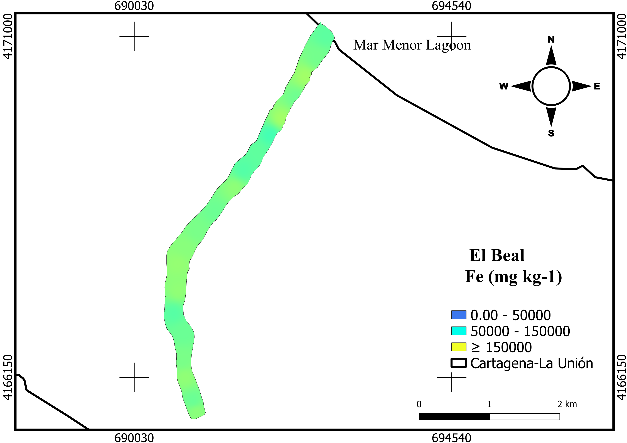 | 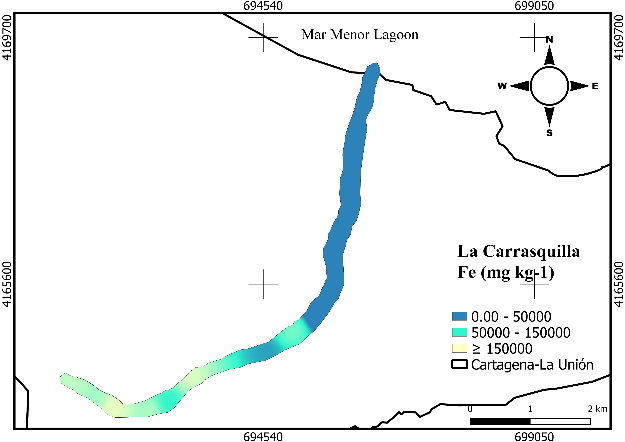 |
| --- | --- |
| 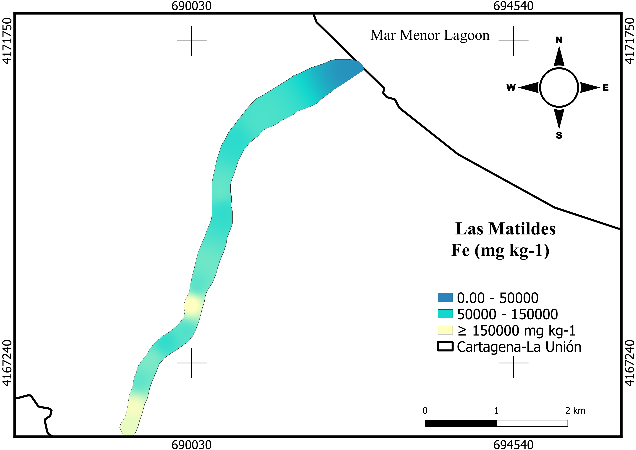 | 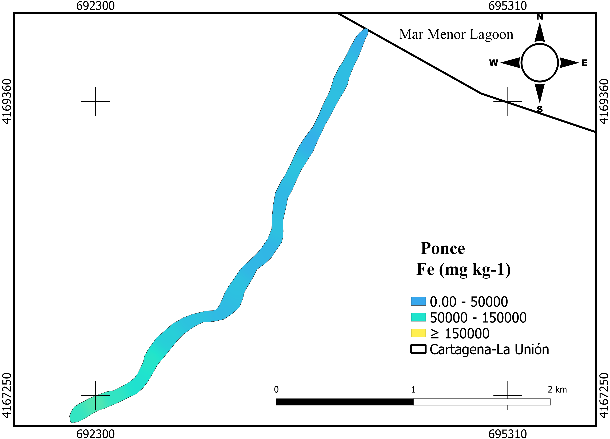 |

**Fig.5** Fe spatial distribution in soil samples from dry riverbeds.

| 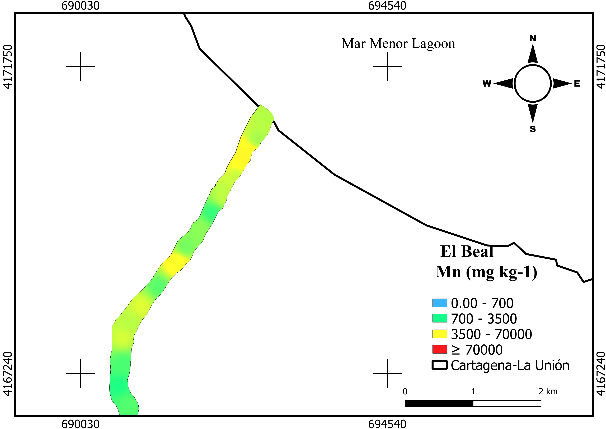 | 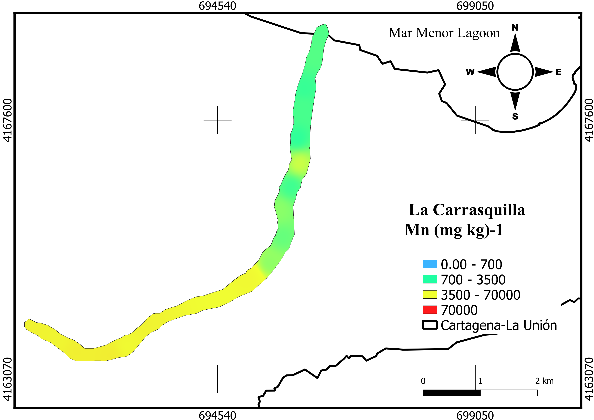 |
| --- | --- |
| 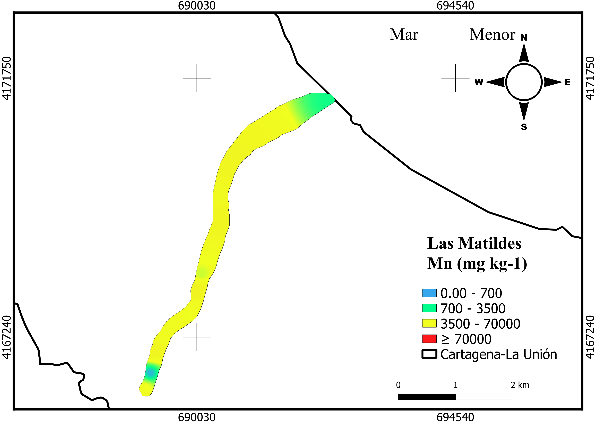 | 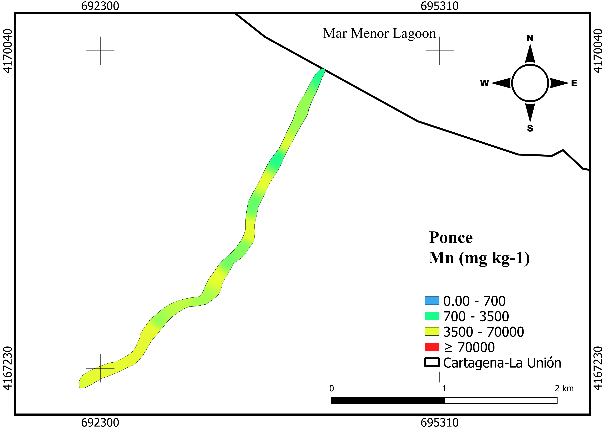 |

**Fig.6** Mn spatial distribution in soil samples from dry riverbeds.

| 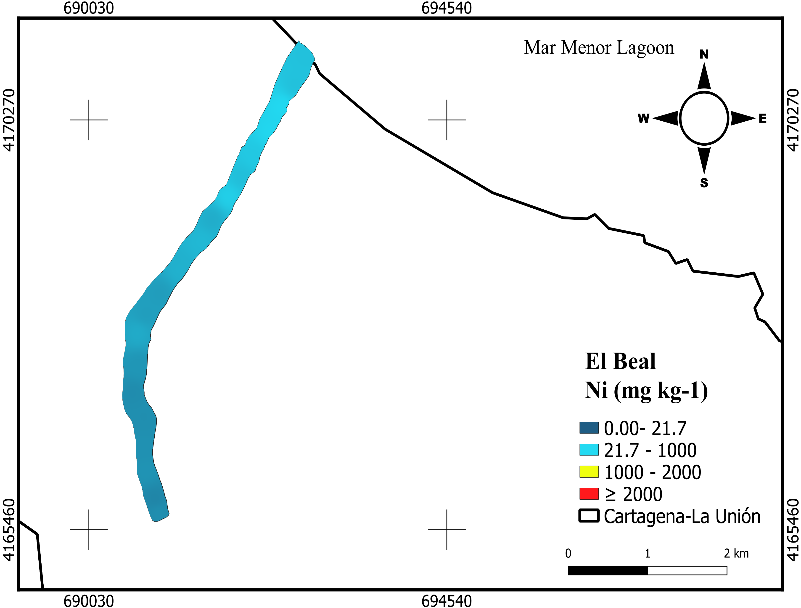 | 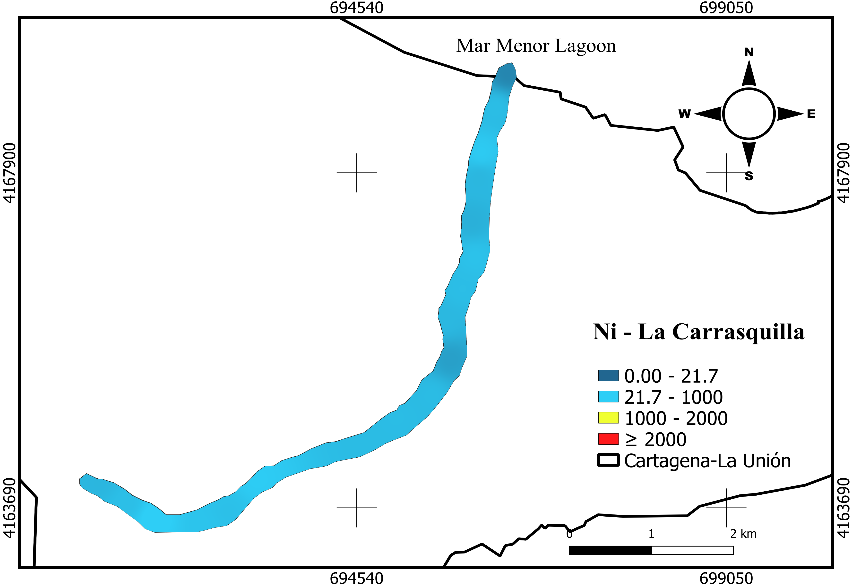 |
| --- | --- |
| 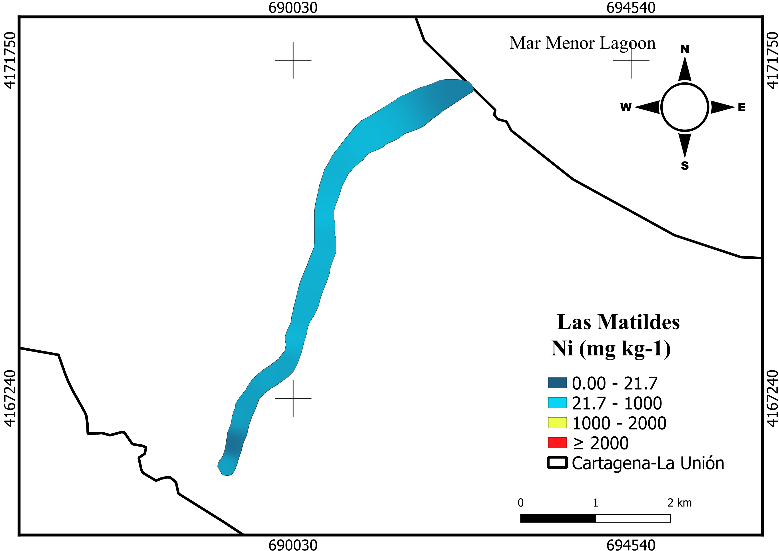 | 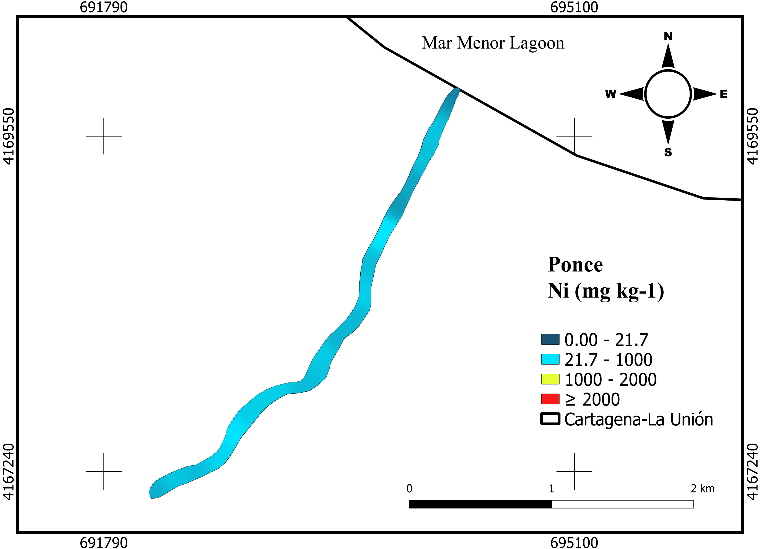 |

**Fig.7** Ni spatial distribution in soil samples from dry riverbeds.

| 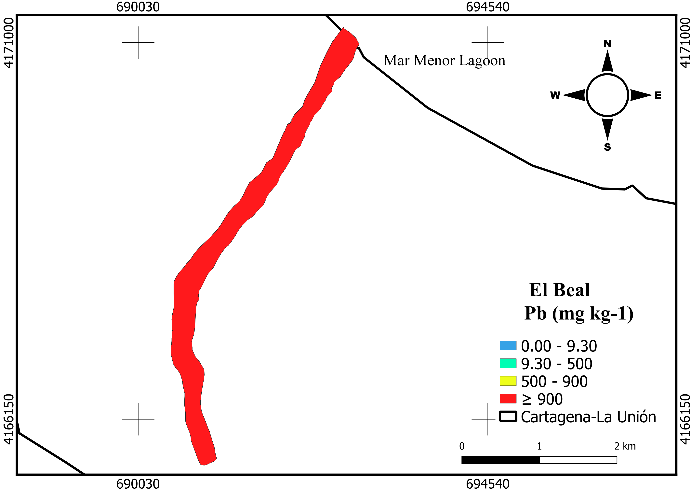 | 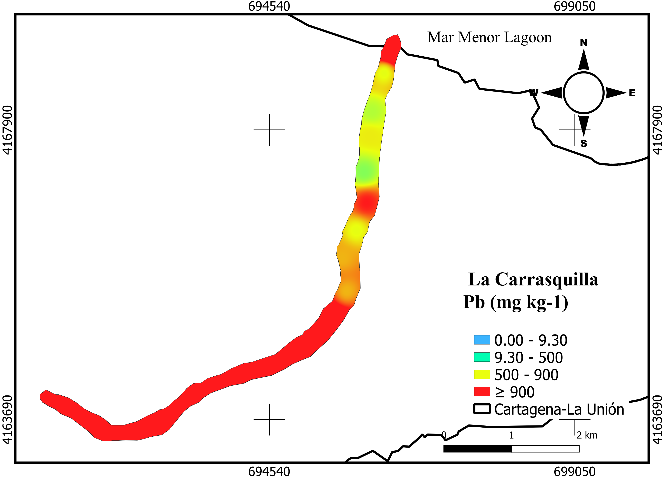 |
| --- | --- |
| 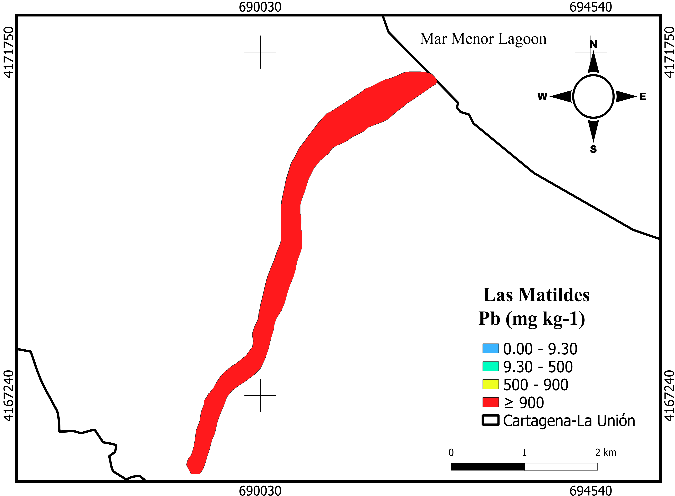 | 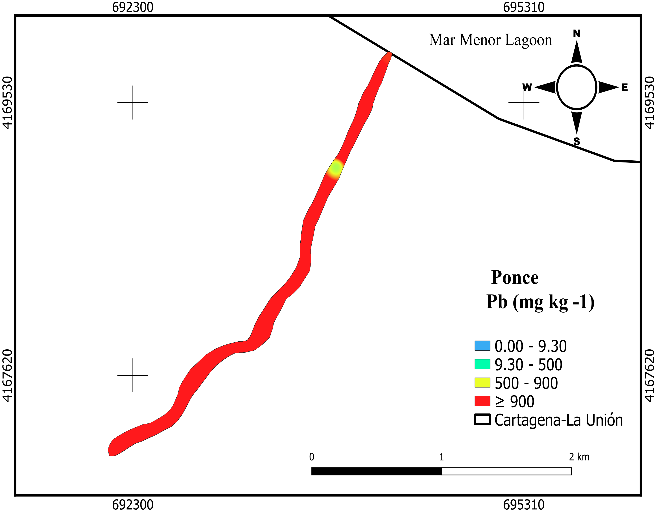 |

**Fig.8** Pb spatial distribution in soil samples from dry riverbeds.

| 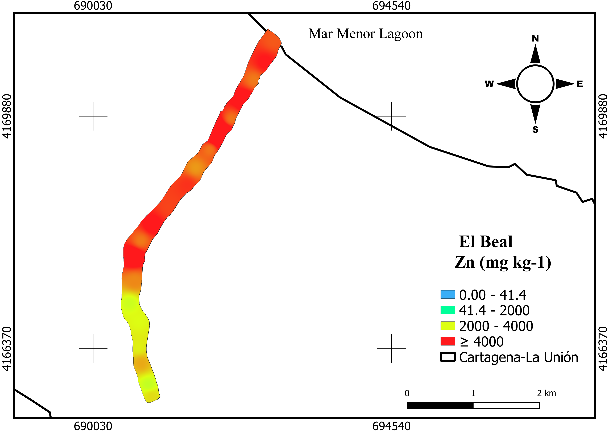 | 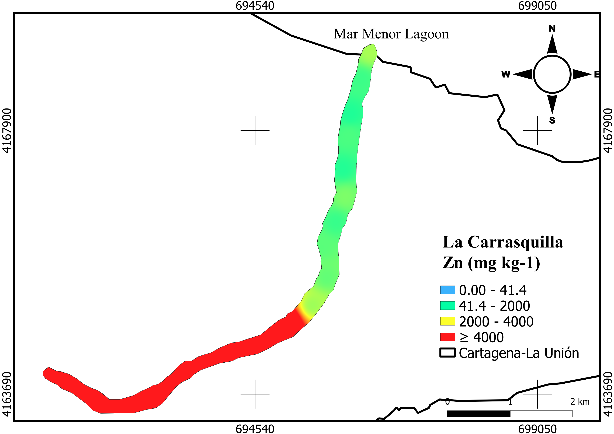 |
| --- | --- |
| 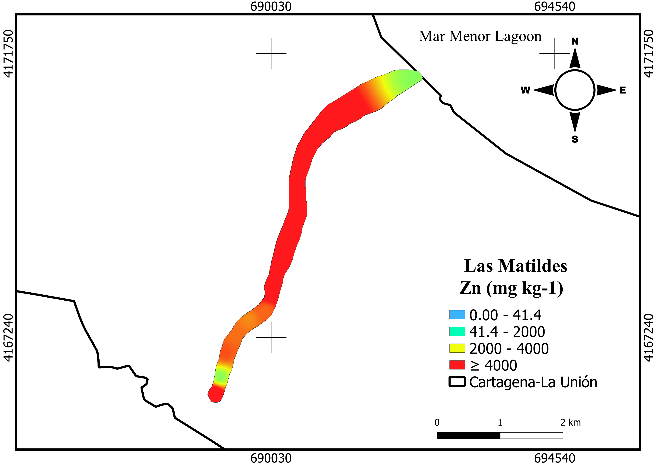 | 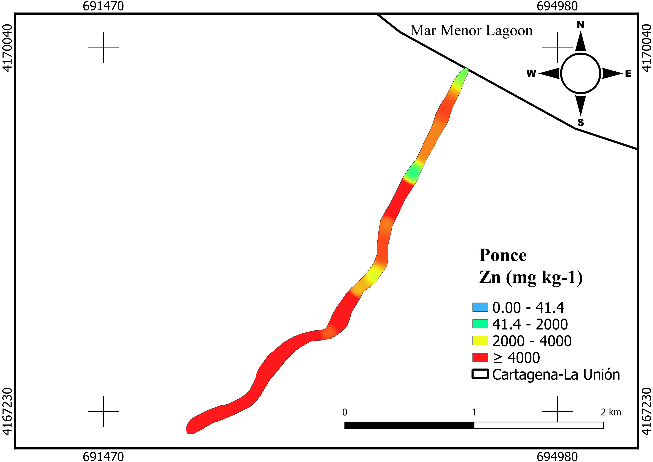 |

**Fig.9** Zn spatial distribution in soil samples from dry riverbeds.

| 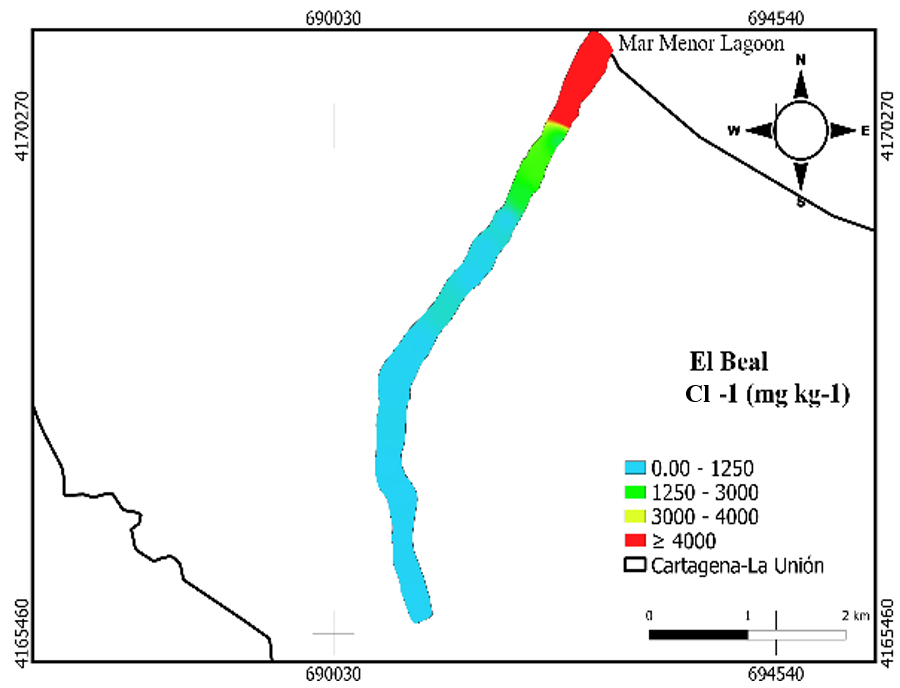 | 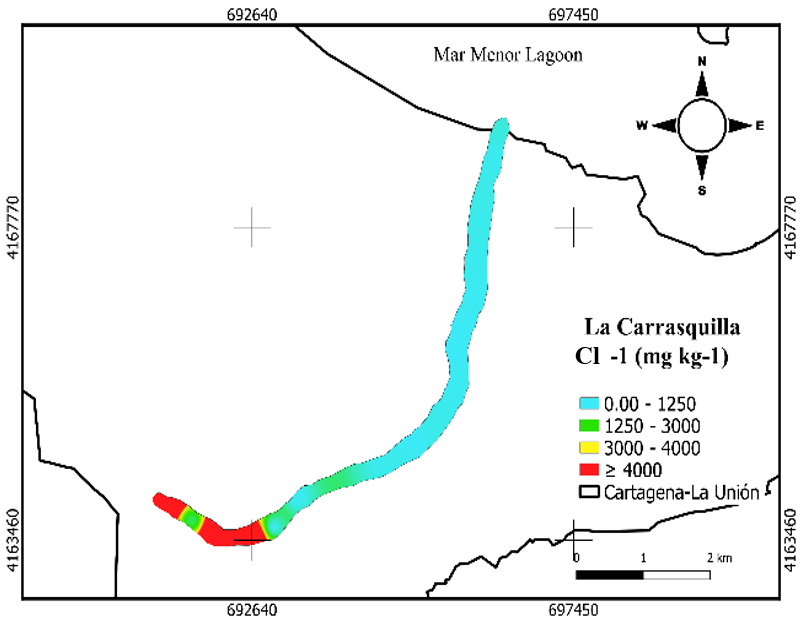 |
| --- | --- |
| 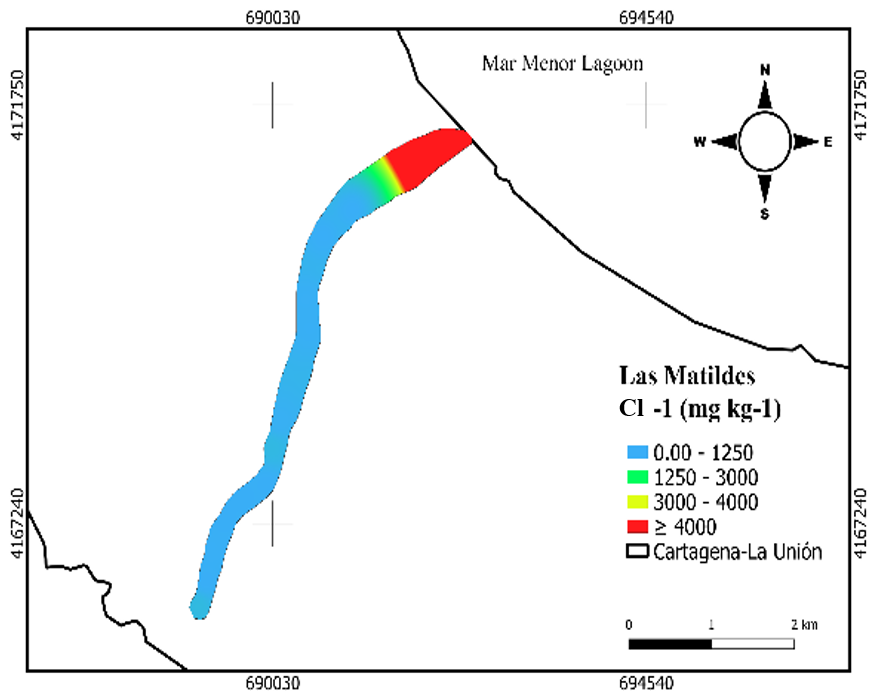 | 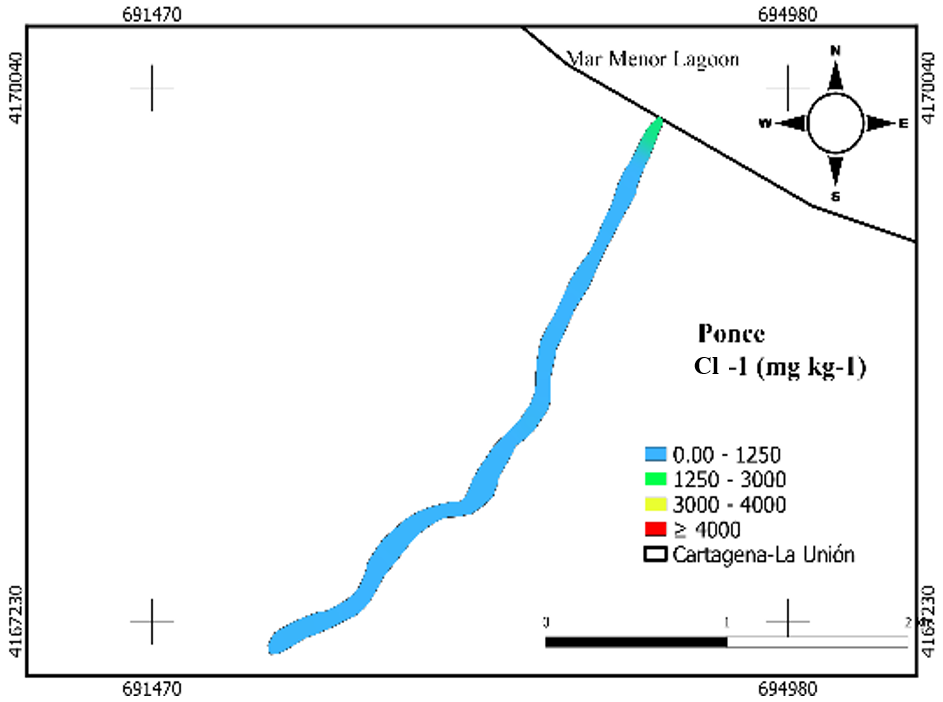 |

**Fig.10** Cl^-^ spatial distribution in soil samples from dry riverbeds.

| 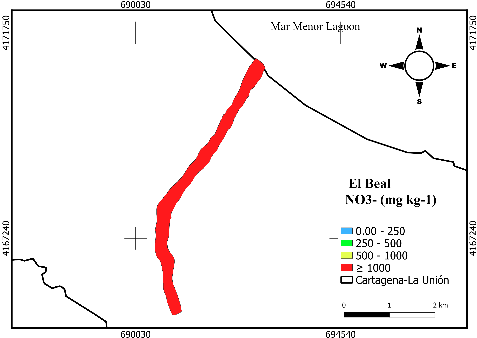 | 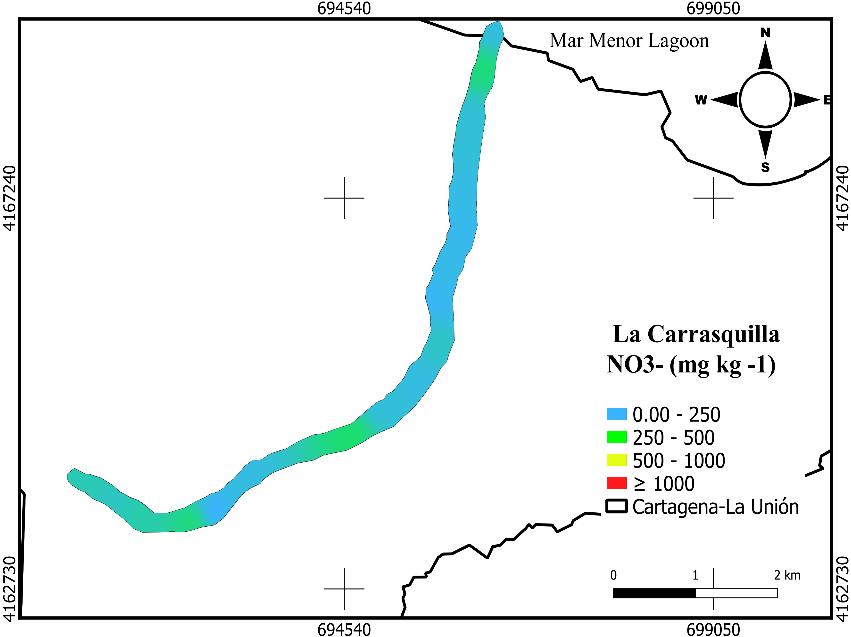 |
| --- | --- |
| 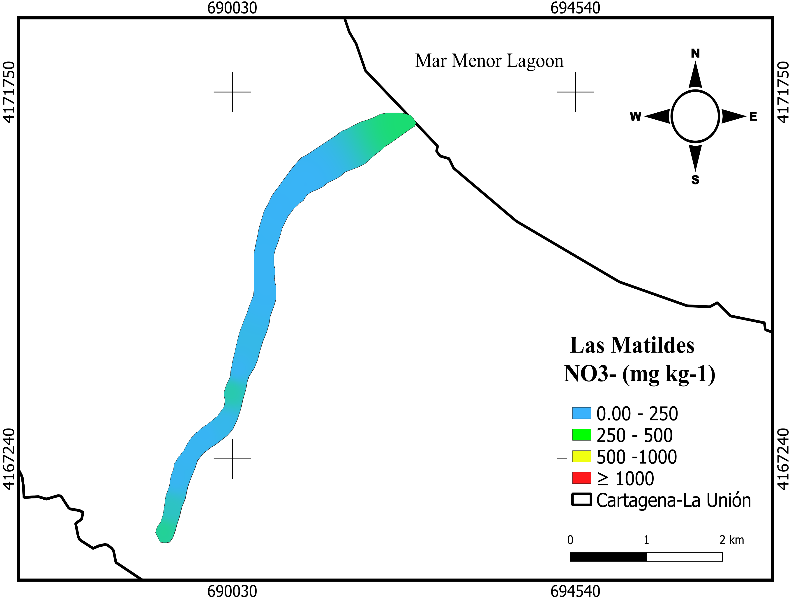 | 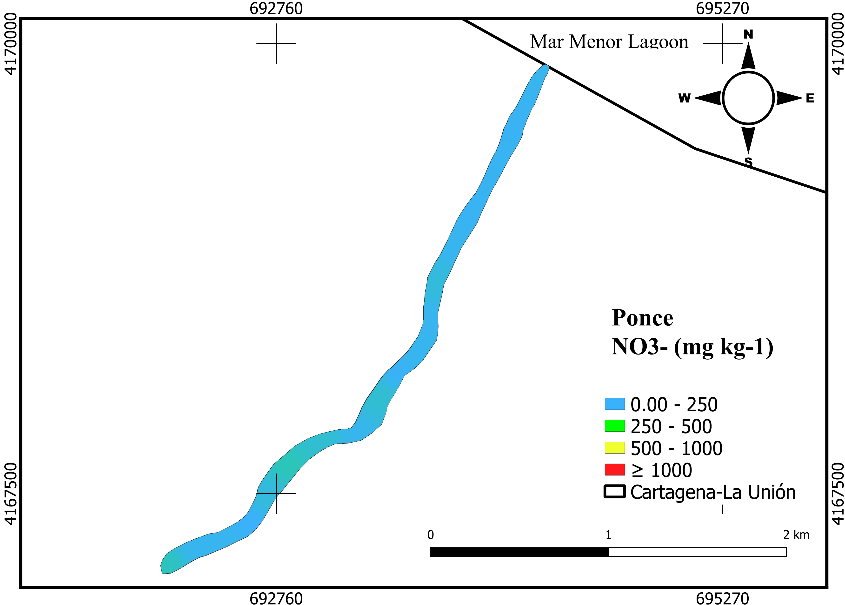 |

**Fig.11** NO_3_^-^ spatial distribution in soil samples from dry riverbeds

| 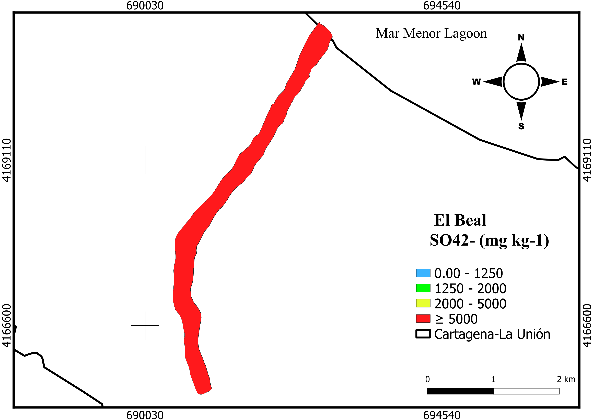 | 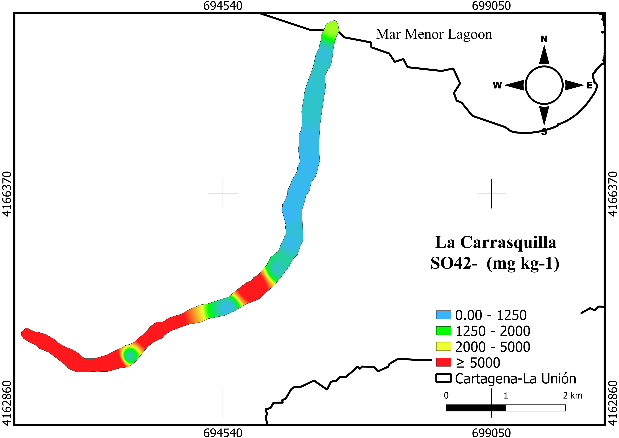 |
| --- | --- |
| 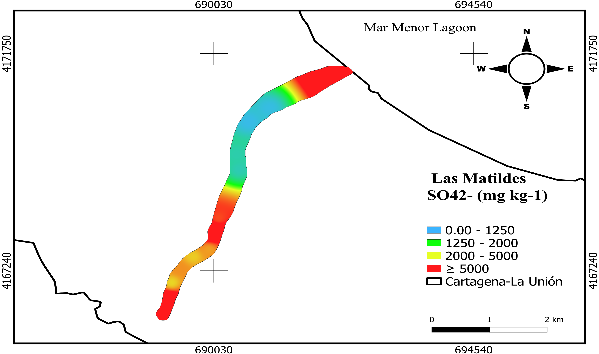 | 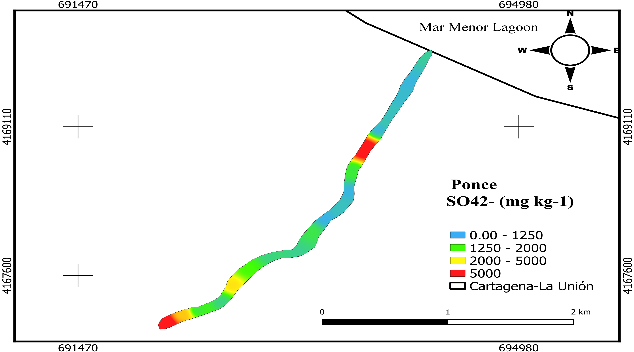 |

**Fig.12** SO_4_^2-^ spatial distribution in soil samples from dry riverbeds

| 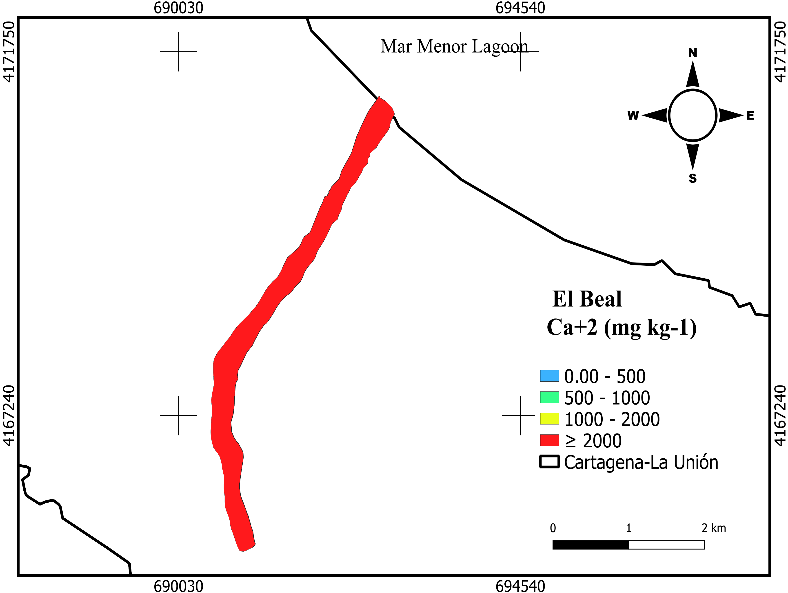 | 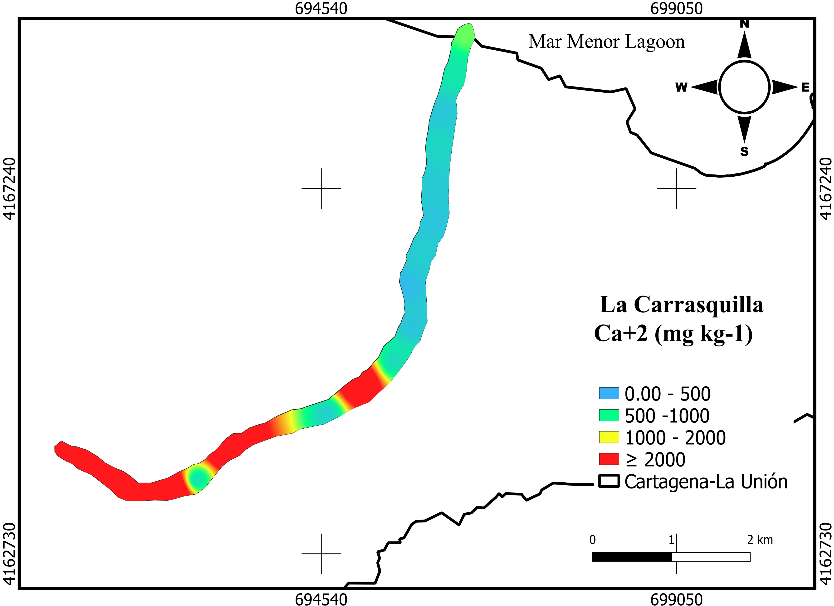 |
| --- | --- |
| 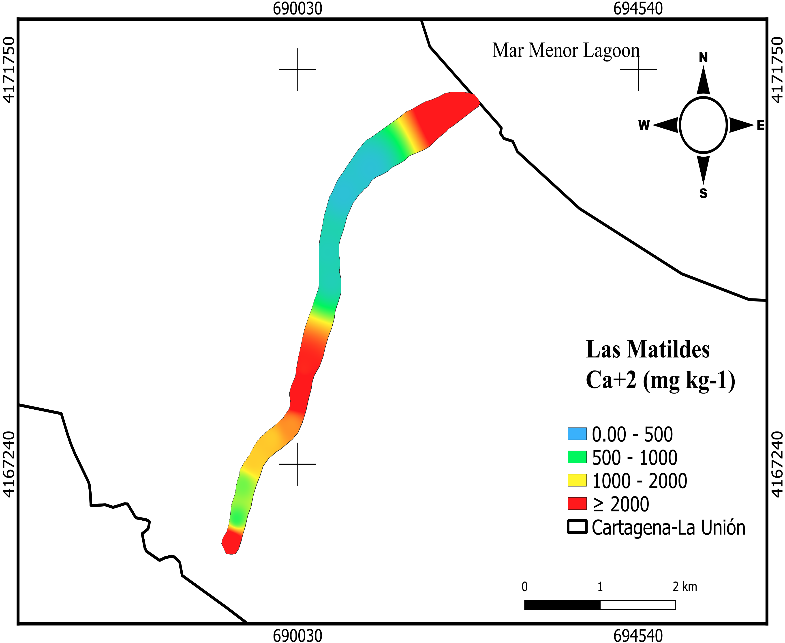 | 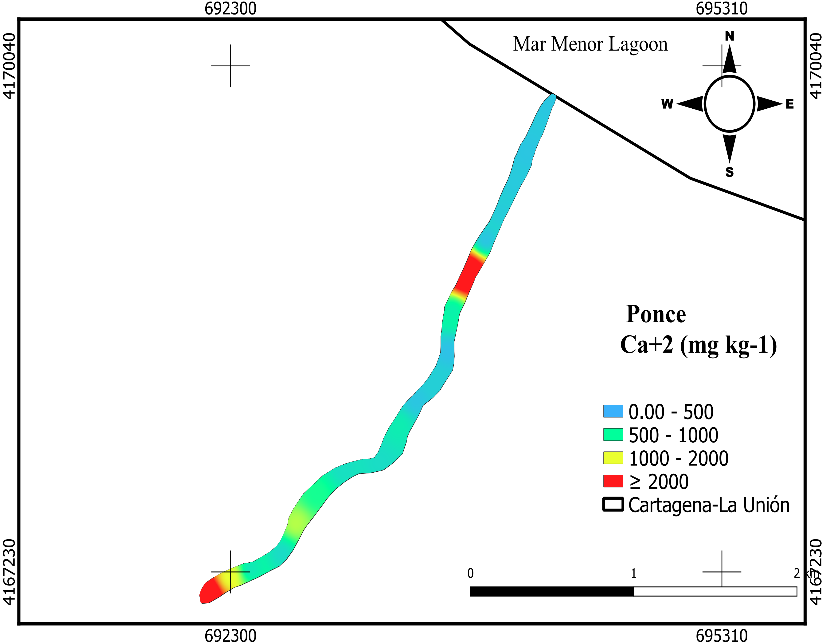 |

**Fig.13** Ca^+2^ spatial distribution in soil samples from dry riverbeds

| 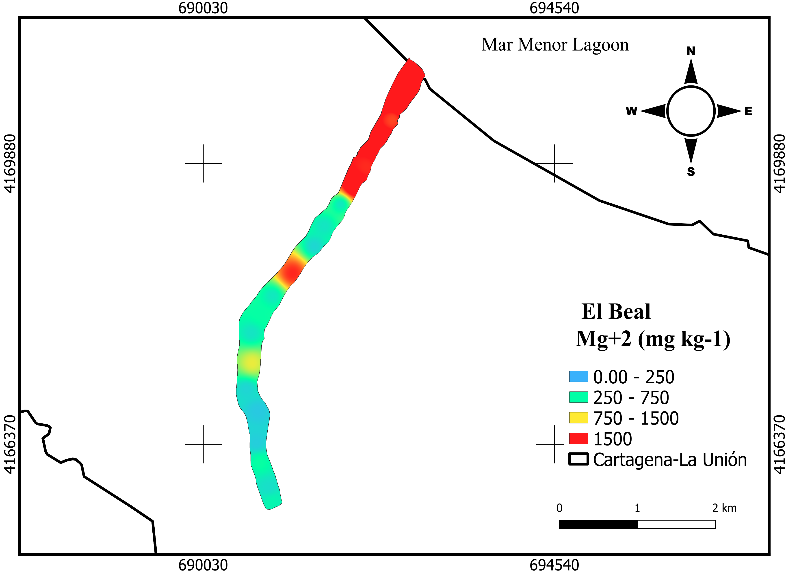 | 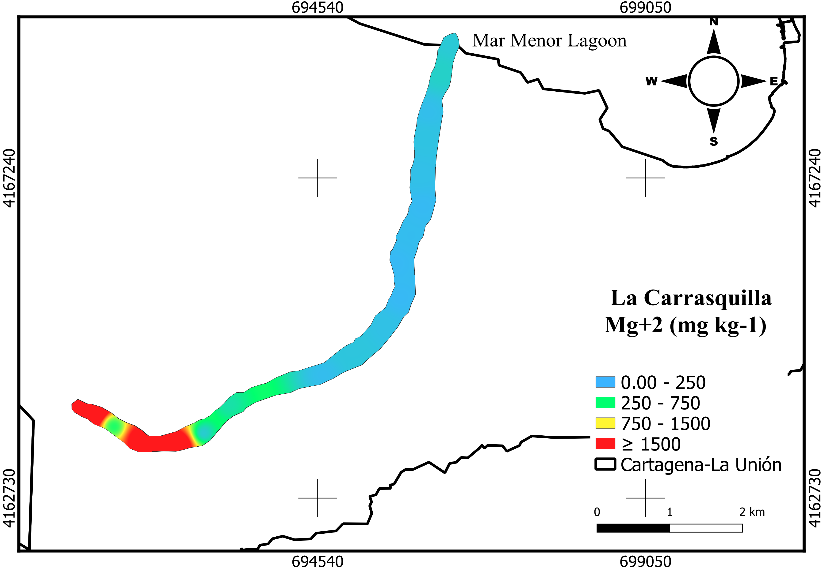 |
| --- | --- |
| 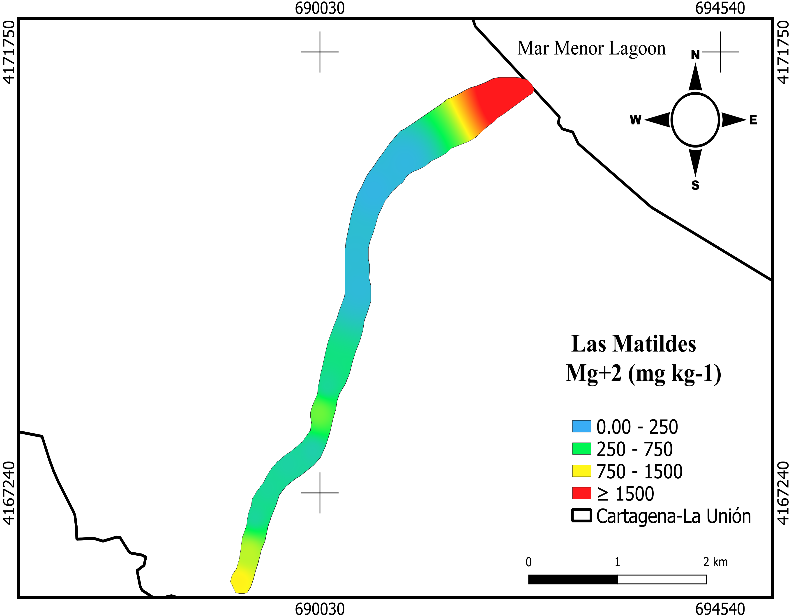 | 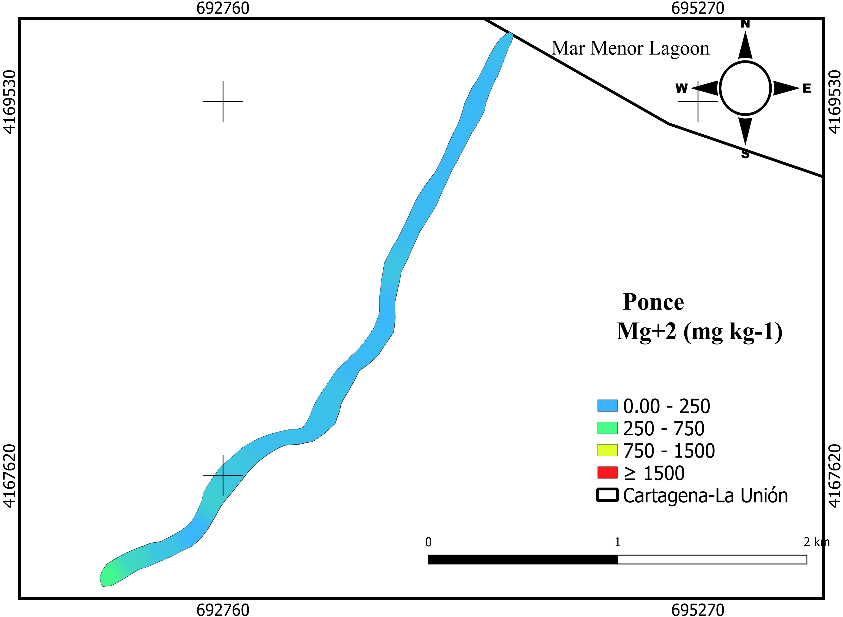 |

**Fig.14** Mg^+2^ spatial distribution in soil samples from dry riverbeds

| 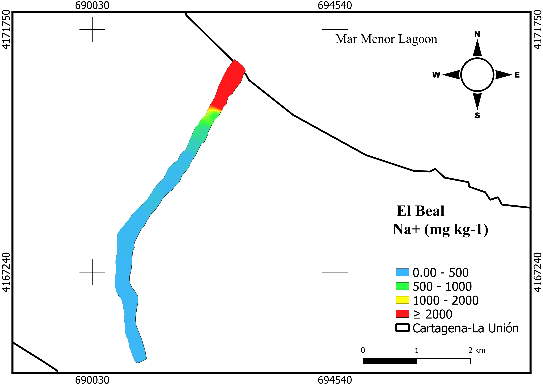 | 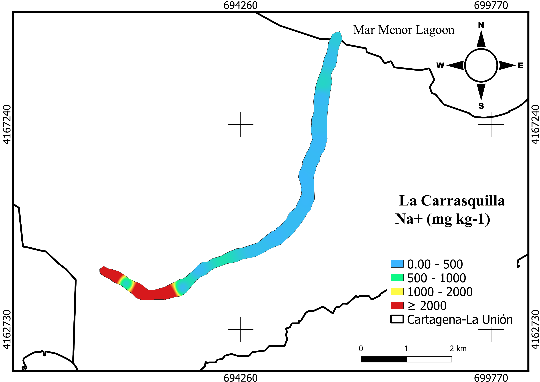 |
| --- | --- |
| 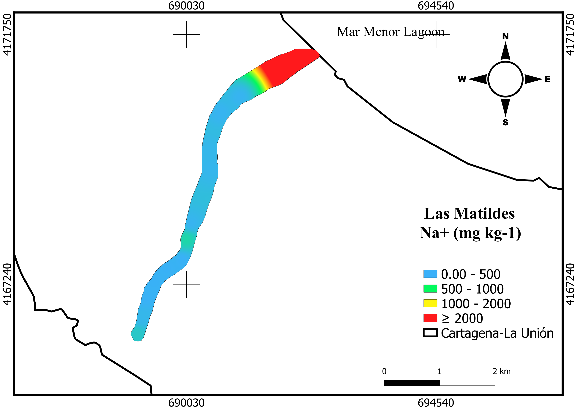 | 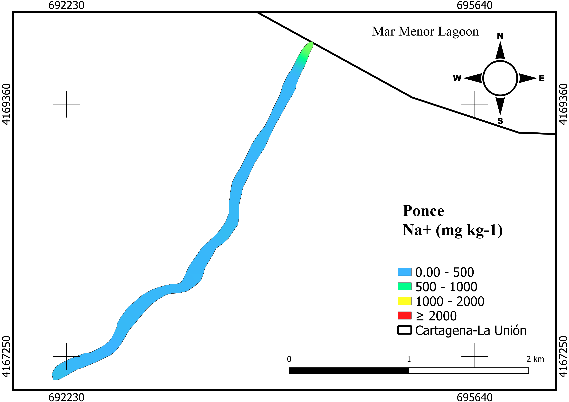 |

**Fig.15** Na^+^ spatial distribution in soil samples from dry riverbeds

| 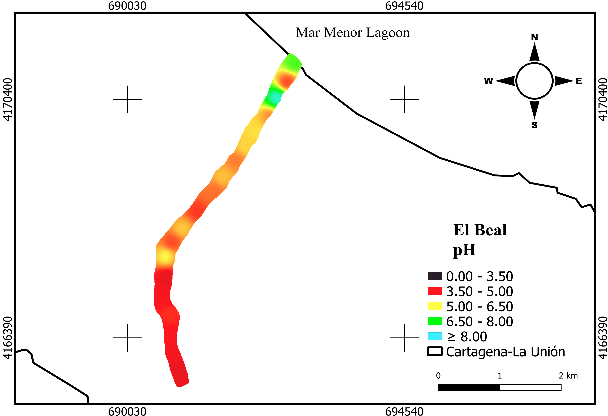 | 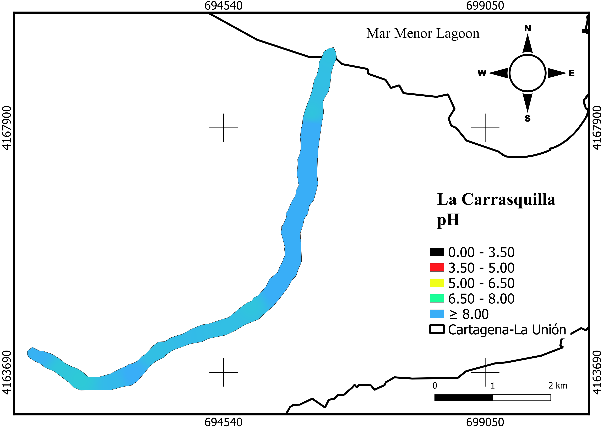 |
| --- | --- |
| 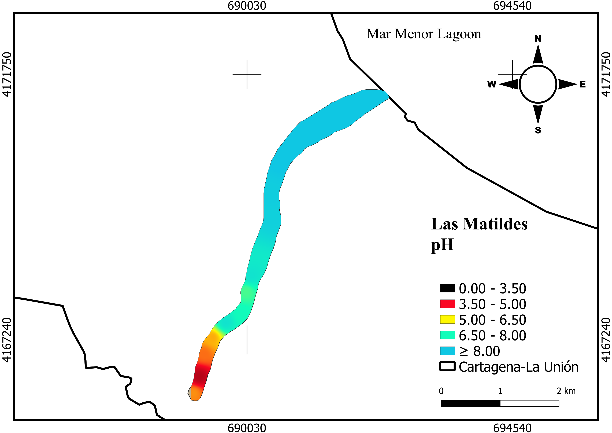 | 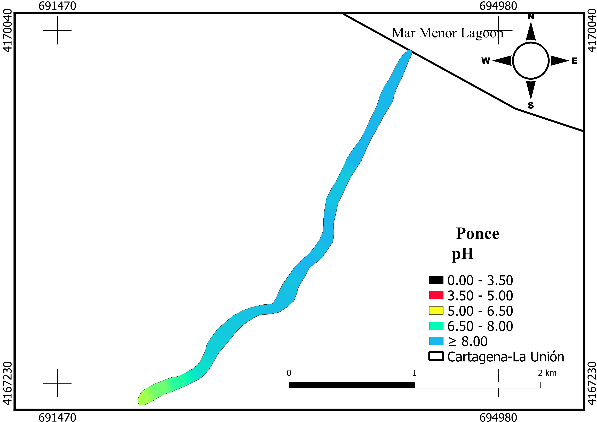 |

**Fig.16** pH spatial distribution in soil samples from dry riverbeds

| 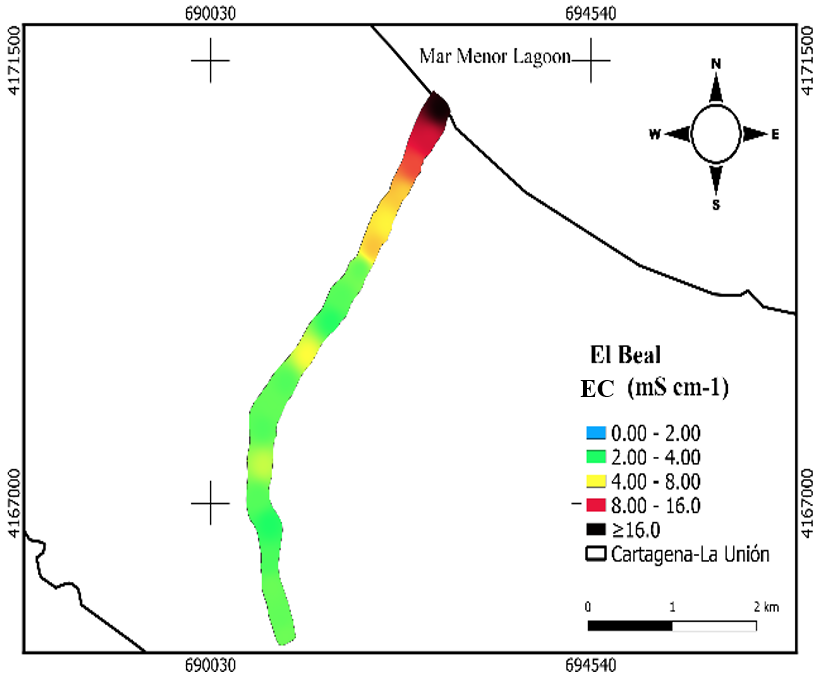 | 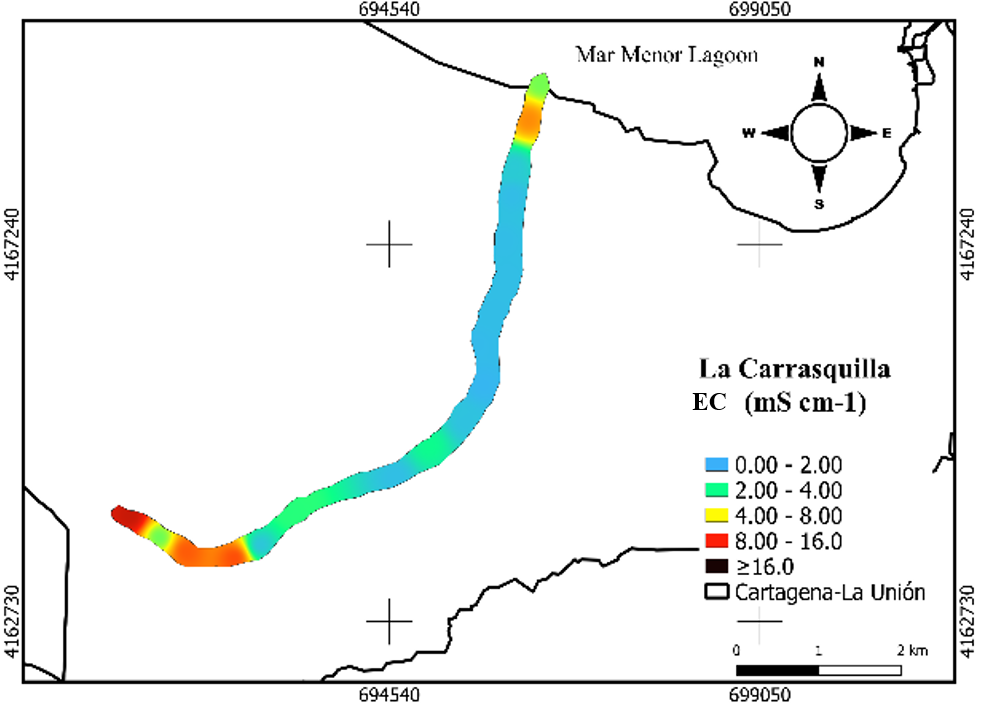 |
| --- | --- |
| 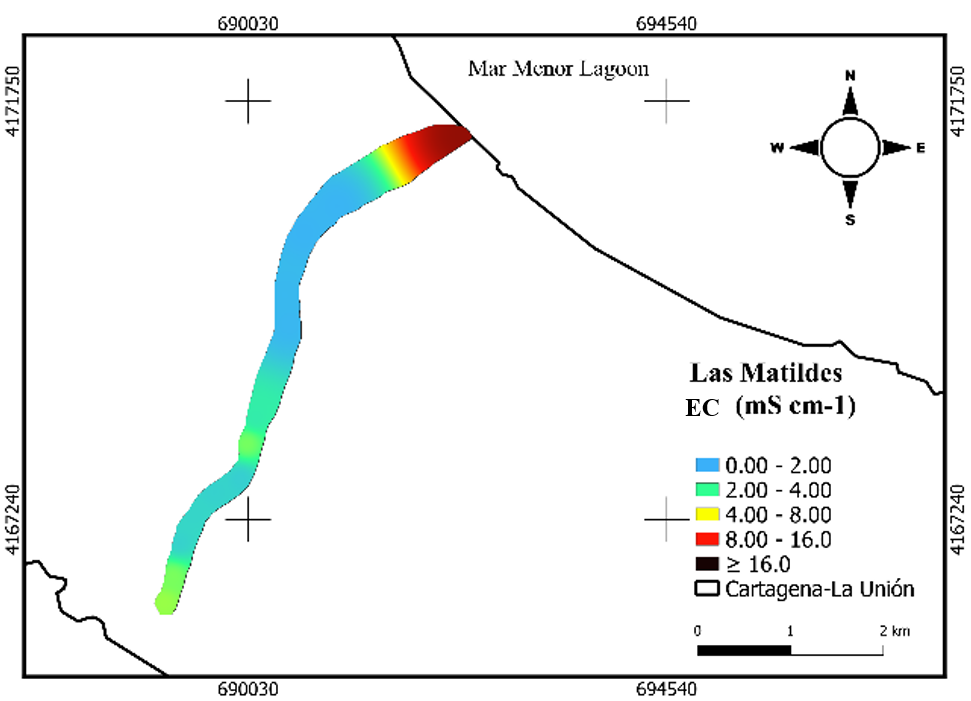 | 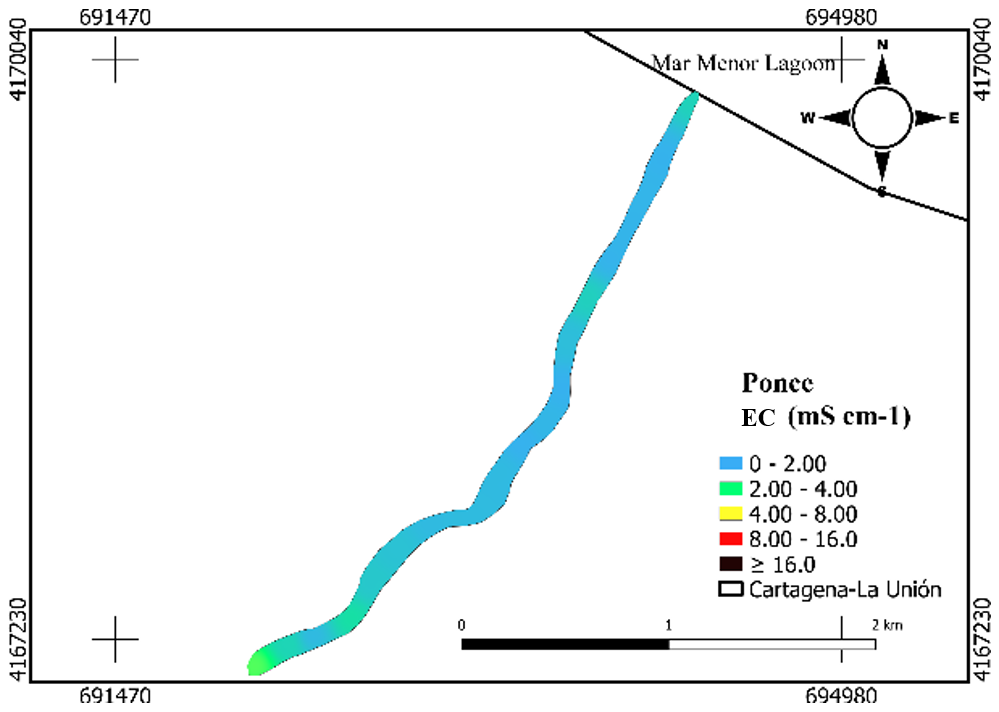 |

**Fig.17** EC spatial distribution in soil samples from dry riverbeds

| 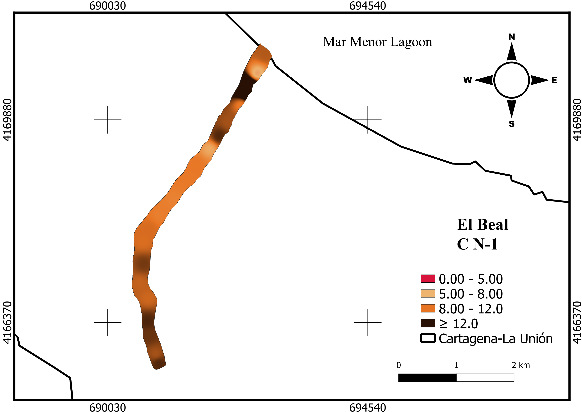 | 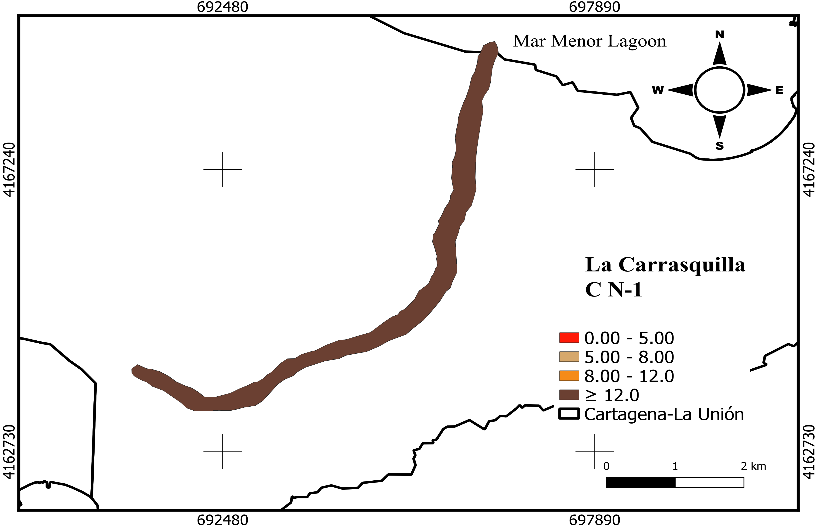 |
| --- | --- |
| 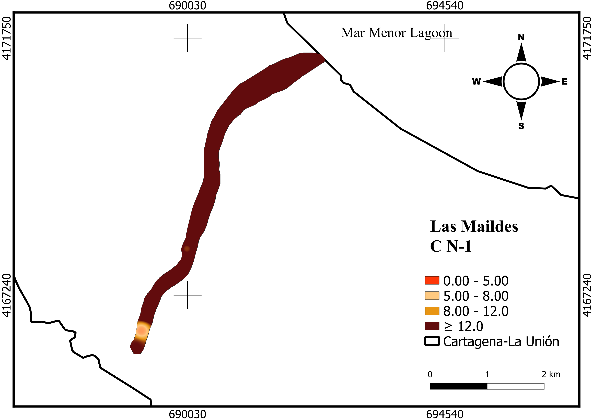 | 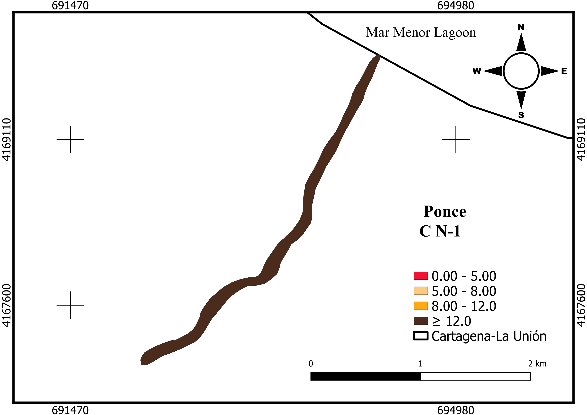 |

**Fig.18** C N^-1^ spatial distribution in soil samples from dry riverbeds
